# Antimicrobial properties and biocompatibility of semi-synthetic carbohydrate-based ionic hydrogels†

**DOI:** 10.1039/d4ra05695g

**Published:** 2024-09-26

**Authors:** Sina Lambrecht, Alina Gazizova, Selin Kara, Johanna Meyer, Stefan Jopp

**Affiliations:** a Department Life, Light & Matter, University of Rostock Albert-Einstein-Str. 25 18059 Rostock Germany stefan.jopp@uni-rostock.de; b Institute of Chemistry, University of Rostock Albert-Einstein-Str. 3a 18059 Rostock Germany; c Institute of Technical Chemistry, Leibniz University Hannover Callinstraße 5 30167 Hannover Germany johanna.meyer@iftc.uni-hannover.de; d Biocatalysis and Bioprocessing Group, Department of Biological and Chemical Engineering, Aarhus University Gustav Wieds Vej 10 8000 Aarhus Denmark

## Abstract

Hydrogels have gained significant interest in the last decades, especially in the medical sector, due to their versatile properties. While hydrogels from naturally occurring polysaccharides (*e.g.* cellulose) are well-known, those produced from polymerizable carbohydrate-based monomers remain underexplored. However, these semi-synthetic hydrogels offer the great advantage of having adjustable properties for customization depending on their application. The objective of this study was to characterize semi-synthetic carbohydrate-based ionic hydrogels produced from GVIM-I (glucosyl vinyl imidazolium iodide). The antimicrobial activity was evaluated using the disk diffusion method, which demonstrated that all samples exhibit inhibitory effects on the growth of *Candida auris*. *In vitro* biocompatibility was determined by cell viability studies with L929 mouse fibroblasts, and a correlation was observed between eluate concentration and cell viability. In particular, the type of initiator system employed for polymerization was found to affect cell viability. The direct contact assessments showed that specific pre-treatments of the hydrogels resulted in higher cell viability than non-treated hydrogels. The results also revealed the impact of crosslinker concentration and type and identified poly(ethylene glycol)diacrylate (PEGDA) 575 as a promising crosslinker for future medical applications. LC-MS analysis of the wash medium identified unreacted GVIM-I as the leached material, which is presumed to be the cause of the observed cytotoxicity. Overall, the study provides valuable insights into the characteristics of GVIM-I based hydrogels and sheds light on the factors that influence their cytotoxicity and potential for medical application.

## Abstract 2

The biocompatibility of carbohydrate-based ionic hydrogels produced from GVIM-I (glucosyl vinyl imidazolium iodide) was evaluated against several microorganisms and L929 cells to determine to applicability of these hydrogels in the medical field.

## Graphical abstract

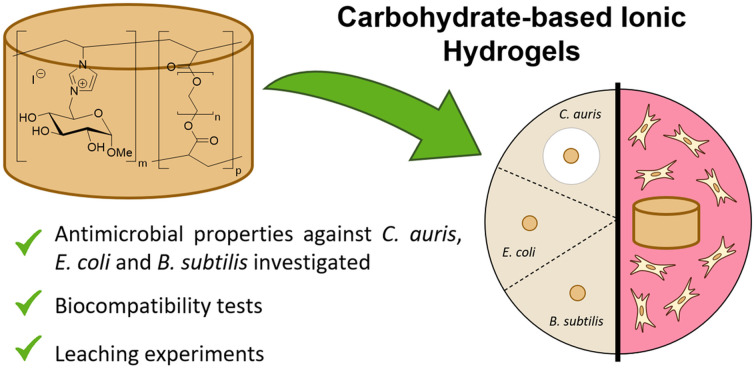

## Introduction

Hydrogels, defined as hydrophilic, three-dimensional polymer networks, have been known since the 1960s^1^ and are widely used in the medical and pharmaceutical industries, *e.g.* as drug delivery systems,^2^ stent coatings,^3,4^ contact lens materials^5^ or for tissue engineering.^6^ Depending on their origin (natural or synthetic), polymer composition, charge, as well as method and degree of crosslinking *etc.*, hydrogels can possess promising properties such as tunable mechanical properties (from stiffness to flexibility),^7,8^ non-toxicity, biocompatibility and even self-healing capabilities.^9^ The aforementioned possible characteristics render hydrogels as promising materials for use in medical applications. In this regard, especially natural hydrogels are of interest due to their high biocompatibility and biodegradability.^10^

Well-known representatives of hydrogels of natural origin are produced from polysaccharides like cellulose, alginate and chitosan, which consist of repeating monosaccharide units and thus also multiple hydroxyl groups.^11–13^ These hydrogels made from sustainable and renewable polysaccharide materials are of great importance in the medical field.^13,14^ Recently, Ding *et al.* showed that non-swelling, injectable chitosan hydrogels are biocompatible with human mesenchymal stem cells (hMSCs) and furthermore exhibit no adverse effects in *in vivo* studies with rats, making them potentially useful as smart biomaterials.^15^ As another example, Ren *et al.* synthesized an injectable hydrogel from quaternized chitosan, gelatin and dopamine for use as drug delivery system for the treatment of Parkinsons's disease.^16^

In comparison to the natural polysaccharide-based hydrogels, the development and biocompatibility of semi-synthetic hydrogels with a carbohydrate component has barely been explored before. The only known previous work in this field, to the best of our knowledge, was published by Goel *et al.*, who recently demonstrated the successful synthesis of biocompatible microporous d-galactose-based hydrogels with a high water uptake of up to 526%. They applied these hydrogels as hydrophilic drug-carrier.^17,18^ Our group has recently studied the synthesis, structural analysis, swelling behavior and degradation of novel semi-synthetic carbohydrate-based ionic hydrogels produced from the cationic starting material GVIM-I (glucosyl vinyl imidazolium iodide) crosslinked with different commercial crosslinkers like polyethylene glycol diacrylate (PEGDA) and *N*′,*N*′-methylenebis(acrylamide) (MBAA).^19^ These hydrogels have a unique combination of properties not found in natural polysaccharide-based hydrogels, as they firstly are polymerized in controllable ratios from a monomer–crosslinker mixture and secondly bear a cationic charge, whereas natural carbohydrates are always neutral or anionic.^11–13^ This cationic charge in the hydrogel potentially enables the binding of anionic drugs such as ibuprofen or naproxen, so that the hydrogel can be used as drug delivery system.^2,20^

Our overall aim is to utilize our novel semi-synthetic carbohydrate-based ionic GVIM-I hydrogels in the biomedical field, *e.g.* in tissue engineering or drug delivery, as previously pointed out. Their low swelling degrees and cationic charge, as investigated in our previous article,^19^ make our GVIM-I hydrogels suitable for these kind of applications.^2,21^ To work towards this goal, we recently also investigated the rheological properties of our GVIM-I hydrogels.^22^

In this work, we want to extend the characterization of our GVIM-I hydrogels in terms of their antimicrobial properties and biocompatibility, as well as investigate the components that leach out of the hydrogels in an aqueous medium.

To determine the antimicrobial activity, we used the gold standard, the disk diffusion method established by Bauer and Kirby *et al.*,^23^ and used *Bacillus subtilis* as Gram-positive, *Escherichia coli* as Gram-negative bacteria strain, as well as *Candida auris* as a widespread yeast. These analyses were performed according to the Clinical Laboratory Standard Institute (CLSI) “Performance Standards for Antimicrobial Disk Susceptibility Tests”.^23^ Furthermore, the *in vitro* biocompatibility was tested by measuring the viability of L929 cells after treatment with hydrogel eluates. Additionally, the viability of cells in direct contact with the GVIM-I hydrogels has also been investigated in this work. These two approaches, in which the cells both come into direct contact with the hydrogels and into contact with the eluates, are mandatory for subsequent applications in the medical field.

## Experimental

### Materials

The chemicals methyl-α-d-glucopyranoside (99%), triphenylphosphine (99%), imidazole (99%), *N*-vinylimidazole (99%), ethylene glycol diacrylate (EGDA) (>90%) and ammonium persulfate (APS) (98%) were obtained from Thermo Fisher Scientific (Darmstadt, Germany). Polyethylene glycol diacrylate (PEGDA) (*M*_n_ = 250, 575 and 700 g mol^−1^), *N*,*N*′-methylene bisacrylamide (MBAA) (99%) and *N*,*N*,*N*′,*N*′-tetramethylethylenediamine (TEMED) (>99%) were supplied by Sigma-Aldrich Chemie GmbH (Taufkirchen, Germany). Iodine (>99.5%) was acquired from Carbolution Chemicals (St. Ingbert, Germany). Lithium phenyl(2,4,6-trimethylbenzoyl)phosphinate (LAP) (>98%) was supplied by TCI (Eschborn, Germany). The solvents THF (99.9%) and ethyl acetate (99.7%) were obtained from Honeywell (Seelze, Germany), the solvents chloroform (>99.8%), methanol (99.8%) and DMF (99.5%) were supplied by Thermo Fisher Scientific (Darmstadt, Germany) and ethanol (99%) was supplied by VWR Chemicals (Darmstadt, Germany) and then diluted to 70% with distilled water. Column chromatography was performed with silica gel (230–400 mesh particle size) obtained from Supelco (Darmstadt, Germany). Additionally, phosphate-buffered saline (PBS; Thermo Fisher Scientific Inc., Waltham, USA) was used.

The starting material 1-(methyl-α-d-glucopyranosid-6-yl)-3-vinylimidazolium iodide (GVIM-I) was synthesized in two steps from methyl-α-d-glucopyranoside as previously published by our group.^19^

### General procedure hydrogel synthesis

The hydrogels were prepared using either the photoinitiator LAP (i) or an initiator system consisting of APS and TEMED (ii). For photopolymerization (i), GVIM-I and LAP were weighed into 1.5 mL Eppendorf reaction tubes, dissolved in PBS (pH = 7.4) and the corresponding amount of crosslinker was added (the exact weights can be found in Tables S1 and S2†). After thorough mixing, the solution was sterile-filtered (Filtropure S, PES, 0.2 μm, Sarstedt AG and Co. KG, Nümbrecht, Germany) using a syringe and poured directly into 6 mm diameter wells of a silicon mold. The samples were then photopolymerized with a UV intensity (*λ* = 365 nm, Biolinker, VILBER, Collégien, France) of 1.2 J cm^2^ and 2.4 J cm^2^, respectively. For radical polymerization (ii),^19^ GVIM-I was weighed into 1.5 mL microcentrifuge plastic tube, dissolved in PBS (pH = 7.4), APS solution and the corresponding amount of crosslinker were added. After thorough mixing, the solution was sterile-filtered and syringed in a sterile Eppendorf reaction tube. TEMED was added, the solution was mixed well and then poured into the silicon mold wells. The gelation took place within a few seconds. Sample names and corresponding components are listed in Table 1.

**Table 1:** List of used crosslinkers, their concentrations, and initiator system, *c*_GVIM-I_ = 1.25 mol L^−1^. All samples with PEGDA as a crosslinker are abbreviated with P, A/T means the initiator system APS and TEMED

Sample name	Crosslinker	*c*_crosslinker_ [mol%]	Initiator
EGDA	EGDA	10	LAP
P250	PEGDA 250	10	LAP
P575 10%	PEGDA 575	10	LAP
P575 15%	PEGDA 575	15	LAP
P575 20%	PEGDA 575	20	LAP
P700	PEGDA 700	10	LAP
MBAA LAP	MBAA	10	LAP
MBAA A/T	MBAA	13	APS/TEMED
P575 A/T	PEGDA 575	10	APS/TEMED

### Antimicrobial activity testing

The antimicrobial activity of the samples was tested using the disk diffusion method, which was established by Bauer and Kirby *et al.* and is considered the gold standard for testing antimicrobial susceptibility.^23^ Tests were performed against some of the most common strains for infections, *Escherichia coli* K-12, *Bacillus subtilis*, and *Candida auris* (WT). These species were stored as glycerol cultures with 20% v/v glycerol at −80 °C. For pre-culture, LB media prepared according to Miller (5 g yeast extract, 10 g peptone, and 10 g NaCl in 1 L ultrapure water) were adjusted to pH 7.0, sterilized by autoclaving, and 10 g per L glucose was added after autoclaving. Bacteria and yeast were cultivated in 150 mL baffled shake flasks at 150 rpm. After the inoculation, the strains were pre-cultured overnight at 35 ± 2 °C before use.

Examinations were performed on Mueller–Hinton agar (for fungal cultures 2% v/v glucose was added), prepared according to the manufacturer's instructions, and poured into 100 mm plates. Bacterial solutions were adjusted to a concentration of 1 to 2 × 10^7^ CFU mL^−1^ (0.5 McFarland Standard, OD_600_ ≈ 0.120) and spread evenly over the entire Mueller–Hinton agar plate, using a sterilized cotton swab soaked in bacteria solution. The samples (hydrogels with a diameter of 6 mm) were placed on the agar plates with sterile forceps. The negative controls were filter paper disks (6 mm in diameter) with 10 μL LB medium, while the positive controls were gentamicin (Roti®Antibiotic Disks Gentamicin (GEN) 10 μg, 6 mm, 50 Units, Carl Roth, Karlsruhe, Germany) for the bacterial strains and amphotericin B (Roti®Antibiotic Disks Amphotericin B (AP) 100 Units, Carl Roth, Karlsruhe, Germany) for the yeast. Bacteria agar plates were incubated for 18 h, and the yeast agar plates were incubated for 24 h at 35 ± 2 °C. The zones of inhibition (ZOI, diameter) were measured in mm. Experiments were performed in triplicate.

### *In vitro* biocompatibility

#### Cell line and culture conditions

The method was adapted from Jopp and Meyer *et al.*,^24^ in short: L929 cells Murine (*Mus musculus*) fibroblasts (L-929, DMSZ No. ACC2) were purchased from Cell Line Service GmbH (Eppelheim, Germany) were routinely cultivated in 175 cm^2^ cell culture flasks (Sarstedt AG and Co. KG, Nümbrecht, Germany) in Dulbecco's Modified Eagle's Medium (DMEM; Sigma-Aldrich Chemie GmbH, Steinheim, Germany), supplemented with 10% fetal calf serum (Sigma-Aldrich Chemie GmbH, Taufkirchen, Germany) as well as 100 U mL^−1^ of penicillin and 100 μg mL^−1^ of streptomycin (penicillin–streptomycin antibiotic solution; Sigma-Aldrich Chemie GmbH, Steinheim, Germany) in a 5% CO_2_ and humidified atmosphere at 37 °C (Heracell 240 incubator, Thermo Fisher Scientific Inc., Waltham, USA). Cells were uncultivated at 70–85% confluency by trypsin/EDTA solution (Sigma-Aldrich Chemie GmbH, Taufkirchen, Germany) treatment after a washing step with phosphate-buffered saline (PBS; Thermo Fisher Scientific Inc., Waltham, USA). Experiments were performed with cells of passage numbers below 34. 24 h prior to the start of an experiment, cells were seeded in 96 well plates (Sarstedt AG and Co. KG, Nümbrecht, Germany) at a density of 8000 cells per well in 200 μL cell culture medium or in 24 well plates (Sarstedt AG and Co. KG, Nümbrecht, Germany) at a density of 50 000 cells per well in 1 mL cell culture medium.

#### Preparation of the eluate for biocompatibility studies

To examine the biocompatibility of the hydrogels, the eluate was prepared according to ISO 10993-12:2021(E) (Biological Evaluation of Medical Devices—Part 12: Sample Preparation and Reference Materials). To obtain the eluate, the hydrogels were incubated for 72 ± 2 h at 37 °C (with a surface area/volume ratio of 3 cm^2^ mL^−1^) in the respective culture media. A control was established by incubating the culture medium for 72 ± 2 h at 37 °C. For each of the samples under investigation, eluate was removed from the hydrogels and sterile filtration was employed to create a stock solution. The stock solutions were subsequently diluted into concentrations of 100%, 10%, 1%, and 0.1%.

#### Preparations of the hydrogels for direct contact biocompatibility studies

As previously stated, hydrogels were synthesized following the aforementioned methodology (i). For direct contact, only hydrogels with PEGDA 575 at a crosslinker concentration of 10% were used. After gelation, the hydrogels were treated in seven different ways, as detailed in Table 2. Each washing step was performed with a ratio of one milliliter of medium to one hydrogel.

**Table 2:** Overview hydrogel treatments

Sample number	Treatment
1	No treatment
2	Washing in DMEM 72 h
3	Washing in DMEM 3 × 24 h
4	Washing in EtOH 24 h and in DMEM 2 × 24 h
5	UV irradiation 1 h
6	UV irradiation 1 h and washing in DMEM 3 × 24 h
7	UV irradiation 1 h, washing in EtOH 24 h and in DMEM 3 × 24 h

### CellTiter-Blue (CTB) viability assay

Cell viability of L929 cells was quantified using the CellTiter-Blue® cell viability assay (Promega GmbH, Mannheim, Germany). This involved the use of cell-free controls for background fluorescence correction and untreated cell controls, in accordance with the manufacturer's instructions. In metabolically active cells, the reduction of blue resazurin to purple, fluorescent active resorufin occurs. The resulting fluorescence intensity is found to be correlated with the number of viable cells. Resorufin formation was monitored using a fluorescence plate reader (Fluoroskan Ascent, Thermo Fisher Scientific Inc., Waltham, USA) with an excitation wavelength of 544 nm and an emission wavelength of 590 nm. L929 cells were cultured in cell culture medium or cell culture medium with varying concentrations of hydrogel eluates for 48 h (approximately 27 500 cells per cm^2^). Afterward, the medium was carefully removed, and 100 μL (96 well plate) or 300 μL (24 well plates) cell culture medium containing 10% CTB stock solution was added to each well. The cells were then incubated until the control fluorescence, which was measured in a plate reader, reached a range of 100–400 relative fluorescence units (RFU). Three biological replicates, each comprising six technical replicates, were analyzed.

### Microscopic analysis

Microscopic imaging of the cells was performed using an IncuCyte S3 Live-Cell Analysis Instrument (Sartorius AG, Göttingen, Germany) prior to and after 24 h and 48 h of treatment with the eluate or hydrogel samples. Phase contrast imaging was conducted using intrinsic auto-exposure function of the IncuCyte software (Sartorius AG, Göttingen, Germany) with the 10× objective.

### Live/dead staining of cells

For the purpose of live/dead staining, the cells were treated for a period of 48 h with the different hydrogel eluate concentrations previously described. Subsequently, the medium was carefully removed from the incubated cells, after which 100 μL (96 well plate) or 300 μL (24 well plate) of cell culture medium containing 5 μM calcein-AM (Sigma-Aldrich Chemie GmbH, München, Deutschland) and 0.125 μg per mL propidium iodide (PI) (Sigma-Aldrich Corporation, St. Louis, MO, USA) was added to each well. After the incubation at 37 °C for 15 min, the samples were analyzed with the BioTek Cytation 5 Cell Imaging Multi-Mode Reader (Agilent, Santa Clara, CA, USA). Imaging was performed in brightfield using the intrinsic auto-exposure function of the Gen5 imaging software (Version 3.10.06, BioTek Instruments GmbH, Bad Friedrichshall, Germany) with a 4× objective. For the detection of dead cells, dyed with PI, in the red channel, the Texas Red filter cube (excitation: 586/15 nm; emission: 647/57 nm) was employed. Conversely, for the detection of calcein-dyed, viable cells, the GFP filter cube (excitation: 469/35 nm, emission: 525/39 nm) was utilized. The following parameters were employed for the detection of PI-stained cells: LED intensity was set to 10, integration time to 1.88 s, and gain to 11. And the following parameters were employed for the detection of calcein-stained cells: LED intensity was set to 5, integration time to 58 ms, and gain to 0.

### Liquid chromatography-mass spectrometry (LC-MS)

A calibration curve was prepared by diluting a GVIM-I solution with a concentration of 1 mg mL^−1^ into a series of standard solutions, with concentrations of 0.2, 0.1, 0.05, 0.025, 0.01, 0.005 and 0.001 mg mL^−1^. Calibration samples were measured on Dionex UltiMate™ 3000 (LC) coupled with LTQ XL™ (MS) with a Phenomenex® Kinetex® C18 column (150 × 2.1 mm, 2.6 μm) at a constant oven temperature of 35 °C with an isocratic eluent composition of 40 : 60 (v/v) MeOH : H_2_O (+0.1% formic acid) (isocratic) and a flow rate of 0.15 mL min^−1^. The detection was conducted *via* MS for the specific mass of the compound.

Hydrogel samples were prepared according to the hydrogel preparation method (i) with PEGDA 575 (10 mol%) as a crosslinker. Subsequently, the hydrogels were placed in 20 mL vials containing pure water (1 mL of water per hydrogel). The hydrogels were stored in water at 37 ± 3 °C for either (A) 72 h or (B) the water was changed after 24 h, 48 h and 72 h. Eluate samples were analyzed using the aforementioned LC-MS system with the following eluent gradient: 40 : 60 (v/v) MeOH : H_2_O (+0.1% formic acid) from 0 min to 3 min, 80 : 20 (v/v) from 10 min to 20 min and again 40 : 60 (v/v) from 30 min to 40 min with a flow rate of 0.15 mL min^−1^. The detection was conducted *via* MS (positive scan mode) for the specific mass of the leached compounds and in parallel *via* MSMS (collision-induced dissociation with a normal collision energy value of 35) for fragments of 271 *m*/*z*.

## Results and discussion

As the first step, carbohydrate-based ionic hydrogels with different compositions were prepared (Table 1). The initiator system of APS and TEMED was used to build on our previous work characterizing the hydrogels, which was focused on the degree of swelling.^19^ Besides the previously used APS and TEMED radical initiator system, we also applied LAP as photoinitiator in this work. It is known from literature that photoinitiators (*e.g.* Irgacure or LAP) exhibit good to very good results in biocompatibility studies with GelMA and PEGDA hydrogels.^25–28^ Xu *et al.* showed that LAP is less cytotoxic than Irgacure 2959 at higher concentrations during 3D printing.^29^ Besides the favorable results of biocompatibility studies, the use of photoinitiators has further advantages. First of all, the polymerization process can be controlled by switching the light source (either UV and/or visible light) on or off. Second of all, photopolymerization takes place under mild conditions (room temperature) and performs very quickly.^30–32^

### Antimicrobial studies

**Fig. 1:**
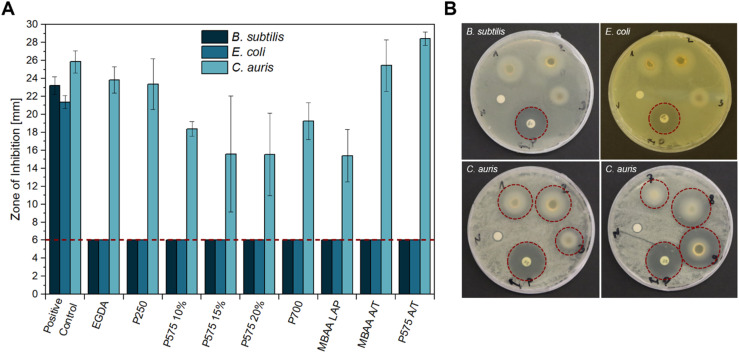
Antimicrobial activity of different hydrogel samples against *B. subtilis*, *E. coli* K12, and *C. auris* (WT) obtained by disk diffusion method. (A) The mean diameter of the zone of inhibition (ZOI) (in mm, including the 6 mm diameter of the disk and sample) of all hydrogels performed in *n* = 3 experiments, error bars indicate +SD. The red dashed line indicates sample size, so that only a ZOI with a bigger size showed an antimicrobial effect. (B) Representative sample agar plates showing the ZOI formed by the hydrogels and the antibiotics (illustrated by the red dashed line, (1) EGDA, (2) P250, (3) P575 10%, (7) MBAA LAP, (8) MBAA APS/TEMED, (9) P575 APS/TEMED, (P) positive control (GEN or AP), (N) negative control (LB medium)). Complete overviews of the disk diffusion tests are given in Fig. S7–S9.†

First, the carbohydrate-based ionic hydrogels with different crosslinkers were tested for their antimicrobial activity towards the Gram-positive strain *B. subtilis*, the Gram-negative strain *E. coli* K-12 and *C. auris* (WT) as a yeast by carrying out disk diffusion assays (Fig. 1). All samples with a diameter of 6 mm were placed on agar plates with the three different microbes. Notably, no inhibitory effect was observed against *B. subtilis* and *E. coli* K-12 in any of the samples. Noteworthy is the whitish circle around the samples in Fig. 1B. This phenomenon could be attributed to the high-water content of the hydrogels, which softens and swells the agar, yet does not affect the microbial growth. The hydrogels are based on the GVIM-I monomer, which has previously been investigated by Jopp and Meyer *et al.* regarding its antimicrobial activity. This previous analysis demonstrated that GVIM-I itself also does not affect bacteria or yeast growth. They demonstrated that GVIM-I, when used at a concentration of 0.1 mol L^−1^, has no inhibitory effect on the growth of *B. subtilis*, *E. coli*, and *C. auris*.^24^ The hydrogels used in this study were prepared with a GVIM-I concentration of 1.25 mol L^−1^.

In the literature, well-known carbohydrate-based materials with antibacterial properties are chitosan-based hydrogels. Chitosan is the second most abundant natural polymer and has antibacterial and antifungal properties.^33–35^ Lahooti *et al.* showed in disk diffusion tests that chitosan–poly(vinyl alcohol) gelatin thyme honey hydrogel films are active against *Staphylococcus aureus* (Gram-positive) and *Pseudomonas aeruginosa* (Gram negative), whereby the higher the chitosan, PVA and honey concentration, the stronger the growth inhibition.^36^ Fan *et al.* and Sajomsang *et al.* showed that quaternary ammonium chitosan hydrogels exhibit strong activity against *S. aureus* and *E. coli*.^37,38^ If silver sulfadiazine or silver nanoparticles are additionally incorporated into chitosan hydrogels, the antibacterial effect against *E. coli* and *S. aureus*, among others, can be further enhanced.^39–41^

The only inhibitory effect of our GVIM-I hydrogel samples was against the yeast *C. auris*. One possible explanation for this could be the different structures of yeast and bacteria. Yeasts (eukaryotes) have no additional peptidoglycan in the cell wall compared to bacteria (prokaryotes).^42^ While many antibacterial agents inhibit the steps that are important for peptidoglycan formation, the essential component of the bacterial cell wall, most antifungal agents act on the function or formation of ergosterol, which is an important component of the fungal cell membrane.^43^ Enache and Cojocaru *et al.* showed that chitosan–nystatin hydrogels have an inhibitory effect against *Candida albicans*, *Candida dubliniensis*, *Candida glabrata* as well as *Candida auris*. They attribute this to the interaction of positively charged amino groups in the chitosan with the negatively charged fungal membrane. The electrostatic attraction of this mechanism causes damage to the cell membrane.^44,45^ Since a positively charged imidazolium is present in the GVIM-I monomer and therefore also in the hydrogel used in this work, this mechanism could also be effective here.

The zone of inhibition (ZOI) of *C. auris* was greatest for the samples of EGDA, MBAA A/T, and PEGDA 575 A/T with 23.83 ± 1.45 mm, 25.43 ± 2.85 mm and 28.45 ± 0.74 mm respectively. PEGDA 575 A/T thus inhibits the growth of *C. auris* stronger than AP as a positive control (ZOI of 25.84 ± 1.22 mm). In comparison to the hydrogels with PEGDA 575 and PEGDA 700, the EGDA and PEGDA 250 hydrogels contain a significantly shorter-chained crosslinker. This can lead to incomplete conversion of monomer and crosslinker and it is assumed that the crosslinker is leached out of the hydrogel during the incubation period. This could potentially inhibit yeast growth due to its toxic properties.^46^ The samples PEGDA 575 15%, PEGDA 575 20%, and PEGDA 700 showed the lowest growth inhibition with a ZOI of 15.59 ± 6.46 mm, 15.54 ± 4.60 mm, and 15.40 ± 2.90 mm respectively.

The final two samples in Fig. 1A were prepared with APS and TEMED as polymerization initiator systems. A comparison of the two samples with their equivalents produced with photoinitiator LAP (P575 10% and MBAA LAP) reveals that the LAP samples cause a significantly smaller ZOI (18.38 ± 0.81 mm and 15.40 ± 2.90 mm). The composition of the reaction solution, which is gelled to form the hydrogel, should be noted here. In the LAP preparations, a concentration of 0.5 wt% to 1.3 wt% of LAP is used in respect to the GVIM-I mass. In case of APS/TEMED, a total concentration of 6.8 wt% to 10.7 wt% is used for the preparations, which results from preliminary investigations.^19^ The exact concentrations for the respective samples can be found in Tables S1 and S2.† The amount of initiator system is therefore higher in APS/TEMED hydrogels, which means that more radicals are present in the system overall. Unreacted radicals (from the initiator, or through further reaction also OH˙ and monomer radicals) can have a negative effect on *C. auris*, whereby its growth is more strongly inhibited.^47–49^

The inhibitory effect against *C. auris* is generally a very valuable property, as it is an invasive pathogenic fungus that is widespread worldwide and poses a threat to human health.^50^ In addition, 93% of *C. auris* strains are resistant to fluconazole, 35% to amphotericin B, and 7% to echinocandins, the common antimycotics used to treat infections with *C. auris*.^51^

### *In vitro* biocompatibility

#### Eluate tests

**Fig. 2:**
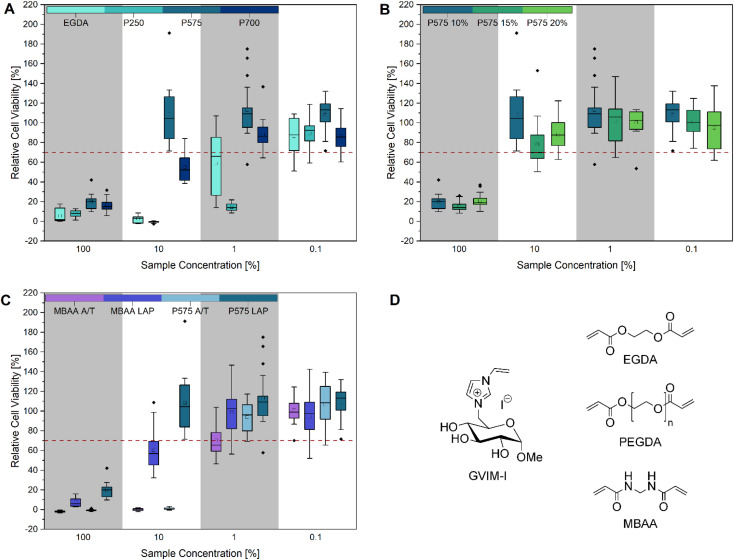
Cell viability after cultivation for 48 h of L929 cells in different concentrated (100%, 10%, 1% and 0.1%) hydrogel eluates: (A) different molar masses of (P)EGDAs (*M*_(EGDA)_ = 170.16 g mol^−1^, *M*_n(P250)_ = 250 g mol^−1^, *M*_n(P575)_ = 575 g mol^−1^, *M*_n(P700)_ = 700 g mol^−1^), (B) different concentrations (10 mol%, 15 mol% and 20 mol%) of PEGDA 575, (C) comparison of MBAA and PEGDA 575 hydrogels with LAP and APS/TEMED as initiator and (D) structure of GVIM-I and the crosslinkers used in this work. The mean value of the wells without cells (background fluorescence) was subtracted from the fluorescence values of the rest of the wells, and the values of treated cultures were normalized to the mean fluorescence of the control cultures. Three biological replicates with six technical replicates each were analyzed, except for P575 10 mol% (10%) and P575 15 mol% (0.1%). These two samples had one biological replication significantly different from the other two and were defined as outliers. Every cell viability of each biological replicate is demonstrated in Fig. S10 and S11.†

A standard protocol for biocompatibility testing, as established by the International Organization for Standardization (ISO), employs the use of L929 cells. This cell line is a trustworthy choice for testing skin contact materials (*e.g.* implants). They are also recommended by several biocompatibility standards (*e.g.* DIN EN ISO 10993-1:2021-05).^52^ In this study, the potential cytotoxicity of hydrogels to L929 cells was investigated using CTB cell viability assay (Fig. 2) and live/dead staining with calcein-AM and PI (Fig. 5B).

As illustrated in Fig. 2, the CTB assay reveals that all samples exhibit cytotoxic properties at the highest concentration tested (100%), which corresponds to the undiluted stock solution of the hydrogel eluates. Fig. 2A compares samples prepared with crosslinkers of the same structure but different molar masses. It can be observed that the samples with EGDA (*M* = 117.15 g mol^−1^) and PEGDA 250 (*M*_n_ = 250 g mol^−1^) exhibit cytotoxic effects at a 10% eluate concentration. In contrast, the samples PEGDA 575 (*M*_n_ = 575 g mol^−1^) and PEGDA 700 (*M*_n_ = 700 g mol^−1^) exhibit significantly higher cell viability, and PEGDA 575 can even be described as biocompatible, as the relative cell viability is over 70%. A reduction in cell viability of more than 30% is considered cytotoxic.^53^ At a 1% eluate concentration, PEGDA 700 also exhibits a similar high cell viability as PEGDA 575. One potential explanation for the high cytotoxicity of EGDA and PEGDA 250 is an incomplete conversion during hydrogel synthesis, resulting in the presence of unreacted monomers, crosslinkers, crosslinker radicals, or initiator radicals in the eluate.^54^ The crosslinker radicals are formed during irradiation with UV light and can cause cell damage due to their cytotoxicity, resulting in low cell viability.^28,55–57^ It can be generally stated that cell viability increases as the eluate concentration decreases. At a concentration of 0.1%, all samples exhibited high cell viability. In Fig. 2B, the influence of varying crosslinker concentrations of PEGDA 575 (10, 15 and 20 mol%) on cell viability was investigated. At 100% eluate concentration, small differences in relative cell viability can be seen with 20.00 ± 7.69%, 15.49 ± 5.28% and 21.71 ± 7.50%, respectively. At 10% eluate concentration, the differences are larger with 108.60 ± 33.31%, 78.21 ± 23.70% and 87.99 ± 14.76% respectively, but not significant due to the high standard deviation. The crosslinker concentration can be used to adjust the stiffness of the hydrogel, as we were recently able to show using this particular example.^22^ Depending on the application, a variable stiffness can be advantageous.^58^

Fig. 2C shows that the use of different initiator systems does have an influence on cell viability. At 100% eluate concentration, the hydrogels photopolymerized with LAP showed slightly higher cell viability (MBAA = 7.30 ± 4.35%, PEGDA 575 = 20.00 ± 7.69%) than the gels with the initiator system APS/TEMED (MBAA = 1.90 ± 0.86%, PEGDA 575 = −0.71 ± 0.83%). This effect was even more pronounced at the eluate concentration of 10%. The MBAA hydrogel eluates with LAP result in a relative cell viability of 60.09 ± 21.23%, while it is −0.15 ± 1.08% for the samples with APS/TEMED. This can also be seen with PEGDA 575. The LAP hydrogel eluates have a relative cell viability of 108.60 ± 33.31% and the APS/TEMED samples only 0.80 ± 0.93%. This trend is also confirmed by the brightfield microscopy images and the live/dead staining (Fig. 5). At the highest concentration of PEGDA 575 LAP and PEGDA 575 APS/TEMED eluate, cells exhibit a rounded morphology and reduced growth compared to the control in the brightfield microscopy pictures, indicating that both samples are cytotoxic at this concentration. However, the live/dead staining revealed a significant difference between the two samples. In contrast to the PEGDA 575 LAP sample, in which some cells were stained with calcein-AM and appeared rounded, the PEGDA 575 APS/TEMED sample exhibited a near absence of viable cells stained with calcein-AM. At a concentration of 10% eluate, the brightfield microscopy image of PEGDA 575 LAP demonstrates a significantly higher number of cells with a normal cell morphology than the PEGDA 575 APS/TEMED sample. The number of living cells stained with calcein-AM is also clearly higher. From the CTB cell viability assay and the live/dead staining results, it can be concluded that the use of LAP is preferable to the use of APS/TEMED as an initiation system for cell contact applications.^59^ Fairbanks *et al.* showed that LAP is well suited for the photo encapsulation of living cells and has advantages (*e.g.* better water solubility and higher polymerization rates) over another well-known photoinitiator Irgacure 2959.^28^ Wilems *et al.* were able to prove that the mESC (mouse embryonic stem cells) and hNSC (human-induced pluripotent stem cell-derived neural stem cells) used exhibited low viabilities after contact with APS/TEMED, making them cytotoxic according to ISO 10993-5. If the initiator is not completely converted during gelation, this can have a negative effect on the cell viability.^53,60^

#### Direct contact tests

**Fig. 3:**
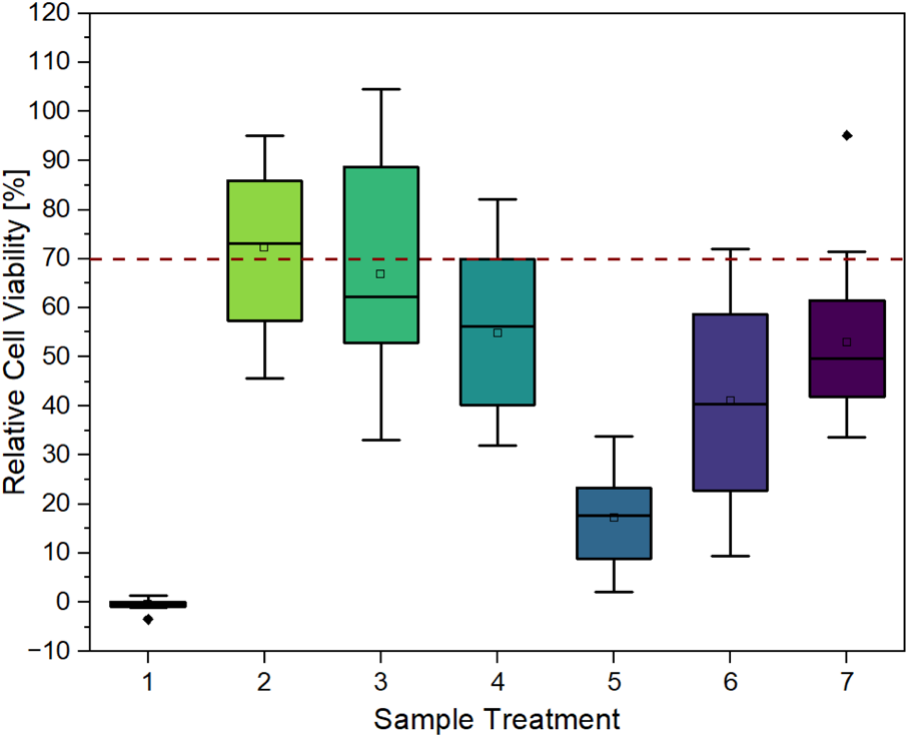
Cell viability after cultivation for 48 h of L929 cells in direct contact with differently treated hydrogels (PEGDA 575, 10 mol%): (1) no treatment, (2) washing in DMEM 72 h, (3) washing in DMEM 3 × 24 h, (4) washing in EtOH (70%) 24 h and in DMEM 2 × 24 h, (5) UV irradiation 1 h, (6) UV irradiation 1 h and washing in DMEM 3 × 24 h, (7) UV irradiation 1 h, washing in EtOH (70%) and in DMEM 3 × 24 h (biological replicates *n* = 3, with 6 technical replicates each).

The previous eluate tests demonstrate the impact of components leached from hydrogels on L929 cells. In this subsequent stage, it is necessary to investigate the growth and morphology of the L929 cells in direct contact with the hydrogels. Therefore, hydrogels were synthesized and treated in accordance with the experimental procedures outlined in Table 2. The treated hydrogels were applied on top of L929 cells that had been incubated for 24 h. After a further 48 h incubation, a CTB cell viability assay (Fig. 3) and live/dead staining with calcein-AM and PI (Fig. 6) were performed.

**Fig. 4:**
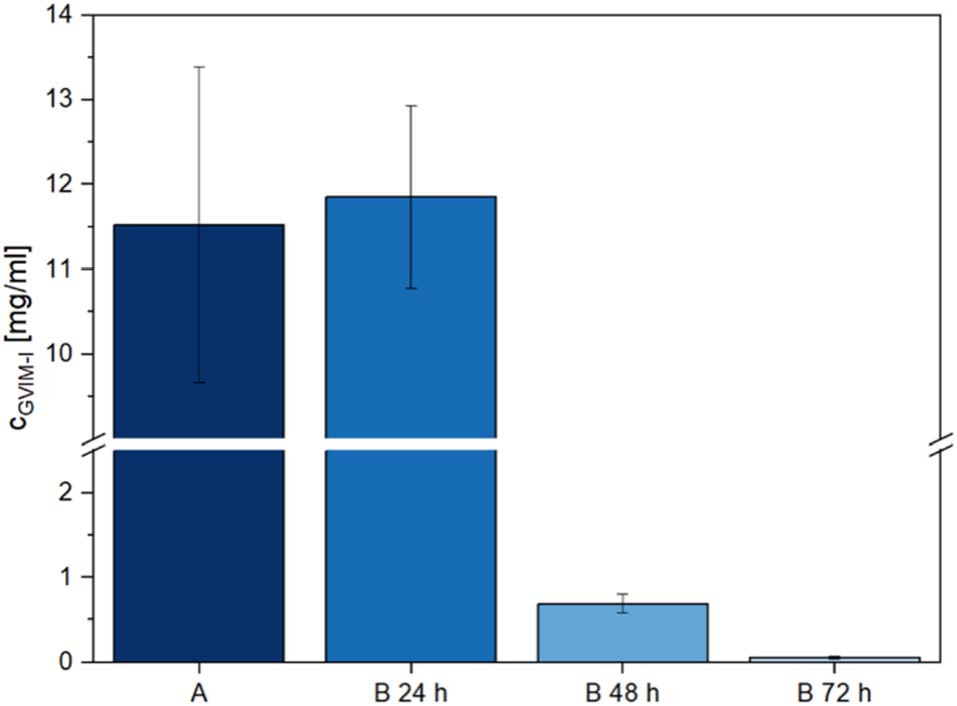
The concentration of leached-out unreacted GVIM-I from hydrogel samples measured by LC-MS. (A) Washing in ultrapure water for 72 h, (B) washing in ultrapure water for 1 × 24 h (B 24 h) 3 × 24 h (B 72 h) (*n* = 3, each sample was measured 3 times *via* LC-MS).

**Fig. 5:**
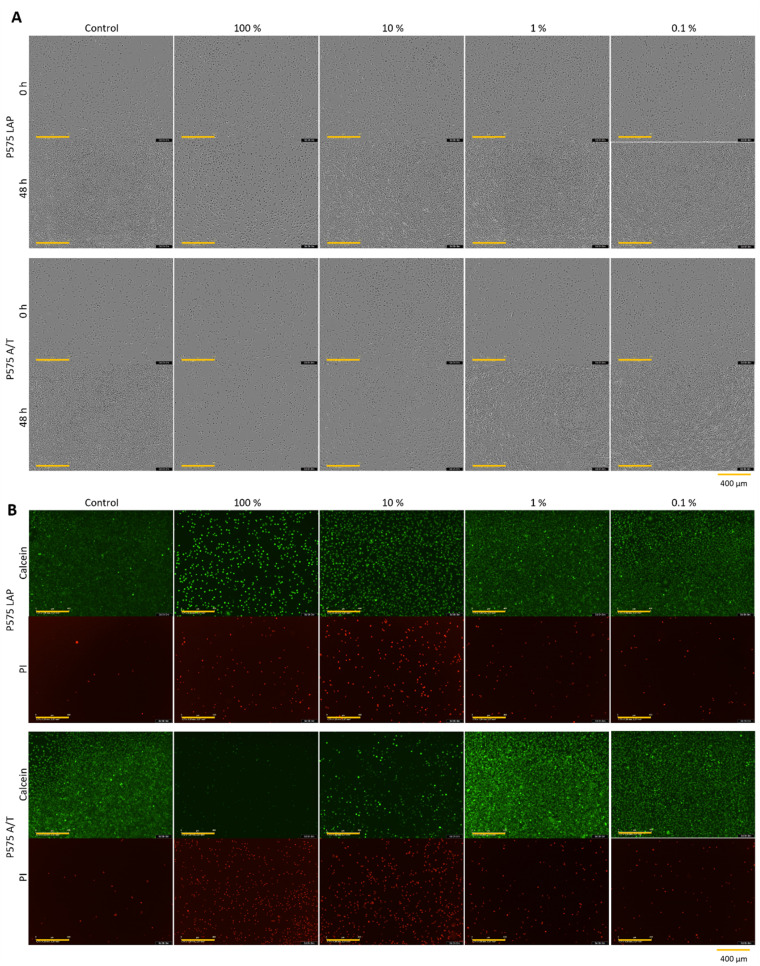
(A) Microscopic images after 0 h and 48 h (yellow scale bar in each image = 400 μm) and (B) calcein-AM/PI staining (yellow scale bar in each image = 400 μm) of L929 cell cultivated for 48 h in different concentrated (100%, 10%, 1% and 0.1%) hydrogel eluates from the hydrogel samples P575 LAP and P575 A/T (which represent hydrogels produced with the 10% PEGDA 575 as crosslinker, polymerized with LAP or APS/TEMED). One representative of each concentration of all samples is demonstrated in Fig. S13, S14 and S16–S18 (see ESI).†

**Fig. 6:**
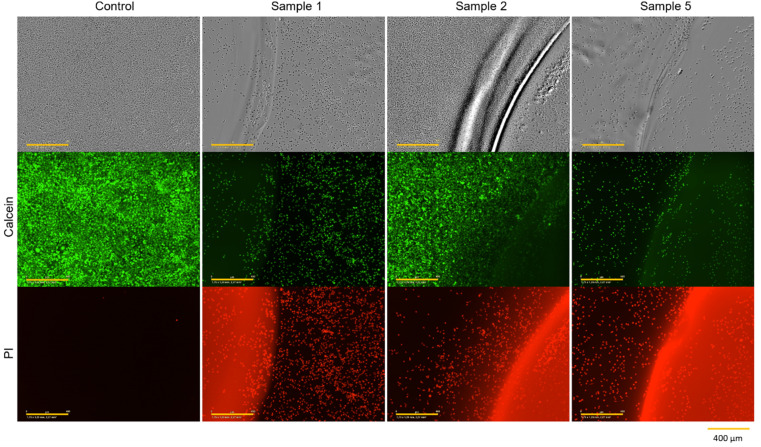
Microscopic images and calcein-AM/PI staining of L929 cells cultivated for 48 h in direct contact with sample 1 (no treatment), sample 2 (washing in DMEM 3 × 72 h) and sample 5 (UV irradiation for 1 h) of PEGDA 575 10 mol% hydrogels (yellow scale bar in each image = 400 μm). One representative of each sample treatment is demonstrated in Fig. S15 and S19 (see ESI).†

Fig. 3 provides clear evidence that contact with untreated hydrogels (sample 1) induces cell death, as indicated by relative cell viability of −0.46 ± 0.99%. This is also confirmed by the microscopy images in Fig. 6. After 48 h of contact, cells exhibited a spherical morphology, indicating cell death. In live/dead staining, calcein stain shows a low number of viable cells, and a high number of dead cells, in comparison to the control.

It can be observed that each treatment leads to a higher relative cell viability than using the hydrogel untreated. The least positive effect was observed in the case of irradiation with UV light for one hour (sample 5) with a relative cell viability of 17.20 ± 9.50%. This treatment was chosen to ensure the highest possible conversion of monomer and crosslinker so that little unreacted monomer and crosslinker as possible is left and can leach out, as these can have cytotoxic effects depending on their concentrations.^24^ As observed in Fig. 6, the low cell viability is reflected in the cell morphology. The cells exhibited a spherical morphology, indicative of cell death. Which is also demonstrated in PI staining, as the majority of cells present are dead.

The combination of UV irradiation for one hour and washing the hydrogels for 3 × 24 h in DMEM at 37 °C (sample 6) resulted in a relative cell viability improvement of approximately 24%. Washing the hydrogels for 3 × 24 h without prior UV irradiation (sample 3) showed a relative cell viability of 66.87 ± 20.86%. When the gels were washed for 1 × 72 h (sample 2), *i.e.* without changing media, relative cell viability was 72.38 ± 15.93%. This high viability is also evident in microscopy images. Fig. 6 shows that the cells have not only survived but have grown. After 48 h, a full lawn can be seen. Live/dead staining confirms this statement. Fig. 6 also indicates that there are more dead cells under and directly next to the hydrogel than further away from the edge of the gel and that cells further away are alive.

In addition to washing with DMEM, ethanol (70%) was also used as a washing medium for samples 4 and 7 (1 × 24 h). However, this did not result in an exceptionally positive effect. Without the ethanol washing step, relative cell viability is higher. In conclusion, different hydrogel treatments lead to an increase in relative cell viability, especially by washing in DMEM.

### LC-MS analysis

The direct contact test results show that washing the hydrogels has a positive effect on relative cell viability. In light of this, it is crucial to ascertain the specific components that are removed from the hydrogels during the washing process, as this process is likely responsible for the pronounced cytotoxicity observed in samples 1 and 5 in Fig. 3. To determine which components had leached out of the samples, hydrogels were prepared following the methodology employed for direct contact tests. They were then washed for 72 h in ultrapure water at 37 ± 2 °C (Fig. 4, A), to simulate sample 2 of Fig. 3. The hydrogels were washed 3 × 24 h in ultrapure water at 37 ± 2 °C (Fig. 4, B 24 h, B 48 h and B 72 h) to simulate sample 3 of Fig. 3. Subsequently, the washing water was then analyzed using LC-MS.

In preliminary investigations, the positive scan of the LC-MS method demonstrated a clear signal at *m*/*z* 271 (Fig. S21A and B†), which precisely corresponds to the mass of the GVIM^+^ monomer ion. In the negative scan mode, the counterion I^−^ was identified at *m*/*z* 126 (Fig. S21C and D†). In all further investigations, the positive scan mode was employed to determine to which extent unreacted GVIM-I is washed out of the hydrogels.

Fig. 4 illustrates that the majority of unreacted GVIM-I is washed out within the first 24 h, with only a very low concentration remaining after the third 24 h period. If the hydrogels are washed only once for 72 h, the total amount of GVIM-I washed out is smaller than with 3 × 24 h, as the concentration gradient is repeatedly increased here by changing the medium. One milliliter of wash medium contains 11.53 ± 1.86 mg of GVIM-I after 72 h of washing. After the first 24 h of washing 11.86 ± 1.08 mg mL^−1^ of GVIM-I was detected, after the second 24 h 0.69 ± 0.11 mg mL^−1^, and after the third time washing 0.06 ± 0.01 mg mL^−1^ was washed out of the hydrogels. To prepare the hydrogels a GVIM-I solution of 500 mg in 1 mL PBS is employed for one batch. An amount of 11.53 ± 1.86 mg washed out from the hydrogels once for 72 h corresponds to 2.3% of the initial GVIM-I amount, whereas 12.61 ± 1.20 mg washed out after washing three times for 24 h and changing the medium in between corresponds to 2.5% GVIM-I of the initially GVIM-I amount. Assuming 2.5% GVIM-I was washed out implies that 97.5% GVIM-I was crosslinked and is involved in hydrogel formation. As a complete conversion of GVIM-I cannot be guaranteed, washing the hydrogels and changing the medium regularly to remove unreacted monomer is a reasonable strategy. This can improve the relative viability of cells in *in vitro* biocompatibility studies.^61^

## Conclusions

In the present study, we successfully investigated our novel carbohydrate-based ionic hydrogels for their antimicrobial properties and *in vitro* biocompatibility. We investigated the properties of the hydrogel eluates on mouse L929 cells as well as the influence of direct contact of the samples on the cells and their growth. In addition, we analyzed the eluates qualitatively and quantitatively by LC-MS.

The hydrogels tested did not affect the growth of the Gram-positive *B. subtilis* and the Gram-negative *E. coli* during disk diffusion tests. However, growth inhibition of *C. auris* was observed in all samples. The choice of crosslinker and initiator system influences the strength of growth inhibition. With the short-chain crosslinkers EGDA and PEGDA 250, the inhibition of *C. auris* growth stronger than with PEGDA 575. It was noticeable that especially the hydrogels with APS/TEMED as the initiator system showed a greater effect than the samples with LAP as initiator.

In our investigation of the eluate toxicity of different hydrogel compositions, we demonstrated that both the choice of crosslinker and initiator system exerts a significant influence on the relative cell viability. The hydrogels with short-chain crosslinkers EGDA and PEGDA 250 showed very low relative cell viabilities with 2.59 ± 3.42% and −0.76 ± 0.83%, respectively, at an eluate concentration of 10%. In contrast, relative cell viabilities of 108.60 ± 33.31% and 54.77 ± 12.96% were achieved for hydrogels with the longer-chain crosslinker PEGDA 575 and PEGDA 700 at the same eluate concentration. Different crosslinker concentrations (10, 15 or 20 mol%) of the same crosslinker (PEDGA 575) exhibited no significant difference in the relative cell viability. The comparison of the polymerization initiators APS/TEMED and LAP revealed that the hydrogel eluates with LAP achieved significantly higher cell viabilities. At 10% eluate concentration, the use of LAP improved cell viability by 60.2% for MBAA and by 32.5% for PEGDA 575.

The direct comparison of the antimicrobial study and the *in vitro* biocompatibility reveals two trends: first, APS/TEMED generally leads to cytotoxic effects and is thus less preferable over the more biocompatible LAP, and second, long-chain crosslinkers (PEGDA 575 and 700) lead to a generally lower cytotoxicity than short-chain crosslinkers (EGDA and PEGDA 250), with PEGDA 575 showing the overall most promising results.

The objective of the direct contact tests was to ascertain the most effective treatment method for enhancing the cell viability of the hydrogels with 10 mol% PEGDA 575 as crosslinker, which are toxic if non-treated. Washing treatments with DMEM and EtOH as well as UV treatment, and combinations thereof, were compared. It was found that for our hydrogels washing treatments are generally preferable over a UV irradiation treatment. The best results were achieved by washing the hydrogels in DMEM for 72 h or 3 × 24 h, leading to relative cell viabilities of 72.38 ± 15.93% and 66.87 ± 20.86%, respectively. Thus, the hydrogels pre-treated with DMEM are biocompatible in the direct contact *in vitro* biocompatibility tests.

LC-MS analysis of the wash water of hydrogels treated with ultrapure water identified unreacted GVIM^+^ in the positive scan mode and found the counter ion I^−^ in the negative scan mode. Quantification revealed that approximately 2.5% of the GVIM-I used in the hydrogel synthesis was found in the wash water, which could possibly be the cause the cytotoxicity in the eluate tests.

The knowledge accumulated in this work enables the development of a standardized production process for our new GVIM-I hydrogels. It is essential to identify synthesis techniques that minimize the quantity of residual starting material. Furthermore, the direct contact experiments showed how important the washing process is for the GVIM-I hydrogels to prepare them for future biomedical applications.

## Data availability

The data supporting this article have been included as part of the ESI.†

## Author contributions

Sina Lambrecht: formal analysis, investigation & writing – original draft; Alina Gazizova: investigation & writing – review; Selin Kara: funding acquisition, writing – review & editing; Stefan Jopp: writing – review & editing; Johanna Meyer: conceptualization, methodology, formal analysis, investigation & writing – original draft. All authors approved the final version of the manuscript.

## Conflicts of interest

There are no conflicts to declare.

## Supplementary Material

RA-014-D4RA05695G-s001

RA-014-D4RA05695G-s002
