# Supplementary material for: Antimicrobial properties and biocompatibility of semi-synthetic carbohydrate-based ionic hydrogels
Source: RSC Adv. 2024 Sep 26;14(42):30719–31. doi: 10.1039/d4ra05695g (PMC11425042; doi:10.1039/d4ra05695g)
Supplement: RA-014-D4RA05695G-s002 [file RA-014-D4RA05695G-s002.pdf]

Supplementary Information (SI) for RSC Advances.
This journal is © The Royal Society of Chemistry 2024

RSC Advances

Supporting Information (SI)

Antimicrobial Properties and Biocompatibility of semi-synthetic Carbohydrate-based Ionic Hydrogels

Sina Lambrecht[a], Alina Gazizova[b], Selin Kara[c,d], Johanna Meyer[c,*], Stefan Jopp[a,*]

[a] Department of Life, Light and Matter, University of Rostock, Albert-Einstein-Str. 25, 18059, Rostock, Germany.

[b] Institute of Chemistry, University of Rostock, Albert-Einstein-Str. 3a, 18059, Rostock, Germany.

[c] Institute of Technical Chemistry, Leibniz University Hannover, Callinstraße 5, 30167 Hannover, Germany.

[d] Biocatalysis and Bioprocessing Group, Department of Biological and Chemical Engineering, Aarhus University, Gustav Wieds Vej 10, 8000 Aarhus, Denmark.

E-mails: johanna.meyer@iftc.uni-hannover.de, stefan.jopp@uni-rostock.de

Table of Contents

1	Synthetic procedures and analytical data	S3
1.1	NMR Spectra	S4
1.2	IR Spectra	S6
1.3	Hydrogel composition	S7
2	Antimicrobial Activity – Disk Diffusion Method	S8
3	Cell viability of L929 cells – CellTiter blue (CTB) viability assay	S11
3.1	Eluate tests	S11
3.2	Direct contact tests	S12
4	Microscopic analysis – Microscopic images of L929 cells	S13
4.1	Eluate tests	S13
4.2	Direct contact tests	S15
5	Cell staining (live/dead) assay – Calcein-AM/PI staining	S16
5.1	Eluate tests	S16
5.2	Direct contact tests	S19
6	LC-MS data	S20

1. Synthetic procedures and analytical data

Synthetic procedure for the synthesis of Methyl-6-iodo-α-D-glucopyranoside (1). Methyl-α-D-glucopyranoside (10 mmol), triphenylphosphine (15.5 mmol), iodine (14.5 mmol), and imidazole (20 mmol) were refluxed in THF (60 mL) for 4 h. The resulting solid was filtered off, the solvent was removed and the product (91 % yield) was obtained after column chromatography ($CHCl_3$/MeOH 12:1). ^{1}H-NMR (300 MHz, DMSO-d_6): δ = 2.87−2.95 (m, 1H); 3.16−3.27 (m, 3H); 3.31 (s, 3H, CH_3); 3.34−3.42 (m, 1H); 3.50−3.57 (m, 1H); 4.54 (d, 1H, ^3J = 3.65 Hz, H-1); 4.78 (d, 1H, ^3J = 6.43 Hz, OH); 4.86 (d, 1H, ^3J = 4.99 Hz, OH); 5.17 (d, 1H, ^3J = 5.83 Hz, OH). ^{13}C-NMR (75 MHz, DMSO-d_6): δ = 9.5 (C-6); 54.6 (CH_3); 70.9, 71.9, 72.7, 74.1 (C-2, C-3, C-4, C-5); 99.8 (C-1).

Synthetic procedure for the quaternization with imidazoles to 1-(Methyl-α-D-glucopyranosid-6-yl)-3-vinylimidazolium iodide (GVIM-I) (2). Methyl-6-iodo-α-D-glucopyranoside (3 mmol) and the *N*-vinylimidazole (5 mmol) were dissolved in DMF (5 mL) and stirred at 95 °C for 24 hours. After cooling down, ethyl acetate (40 mL) was added and the flask was stored in a fridge overnight. The solvent was decanted and the precipitated solid was repeatedly washed with ethyl acetate and dried under a high vacuum to achieve the product, yielding in a light-brown solid (73 % yield). ^{1}H-NMR (250 MHz, D_2O): δ = 3.25 (s, 3 H, OCH_3); 3.24–3.28 (m, 1H, H-4); 3.58 (dd, 1H, ^3J = 9.77 Hz, ^3J = 3.77 Hz, H-2); 3.66–3.75 (m, 1H, H-3); 3.95 (ddd, 1H, ^3J = 9.96 Hz, ^3J = 7.47 Hz, ^3J = 2.46 Hz, H-5); 4.50 (dd, 1H, ^2J = 14.55 Hz, ^3J = 7.38 Hz, H-6a); 4.70 (dd, 1H, ^2J = 14.55 Hz, ^3J = 2.55, H-6b); 4.85 (d, 1H, ^3J = 3.77 Hz, H-1); 5.49 (dd, 1H, ^3J = 8.68 Hz, ^2J = 2.84 Hz, Vinyl-CH_2); 5.86 (dd, 1H, ^3J = 15.58 Hz, ^2J = , Vinyl-CH_2); 7.2 (dd, 1H, ^3J = 15.58 Hz, ^3J = 8.70 Hz, Vinyl-CH); 7.70 (d, 1H, ^3J = 2.09 Hz, H_{Ar}); 7.86 (d, 1H, ^3J = 2.11 Hz, H_{Ar}); 9.16 (s, 1H, H_{Ar}). ^{13}C-NMR (125 MHz, D_2O): δ = 50.2 (C-6); 55.1 (OCH_3); 69.2 (C-5); 70.5 (C-4); 71.0 (C-2); 72.8 (C-3); 99.3 (C-1); 109.8 (Vinyl-CH_2); 119.4, 123.8 (CH_{Ar}); 123.8 (Vinyl-CH); 135.0 (CH_{Ar}).

1.1 NMR Spectra

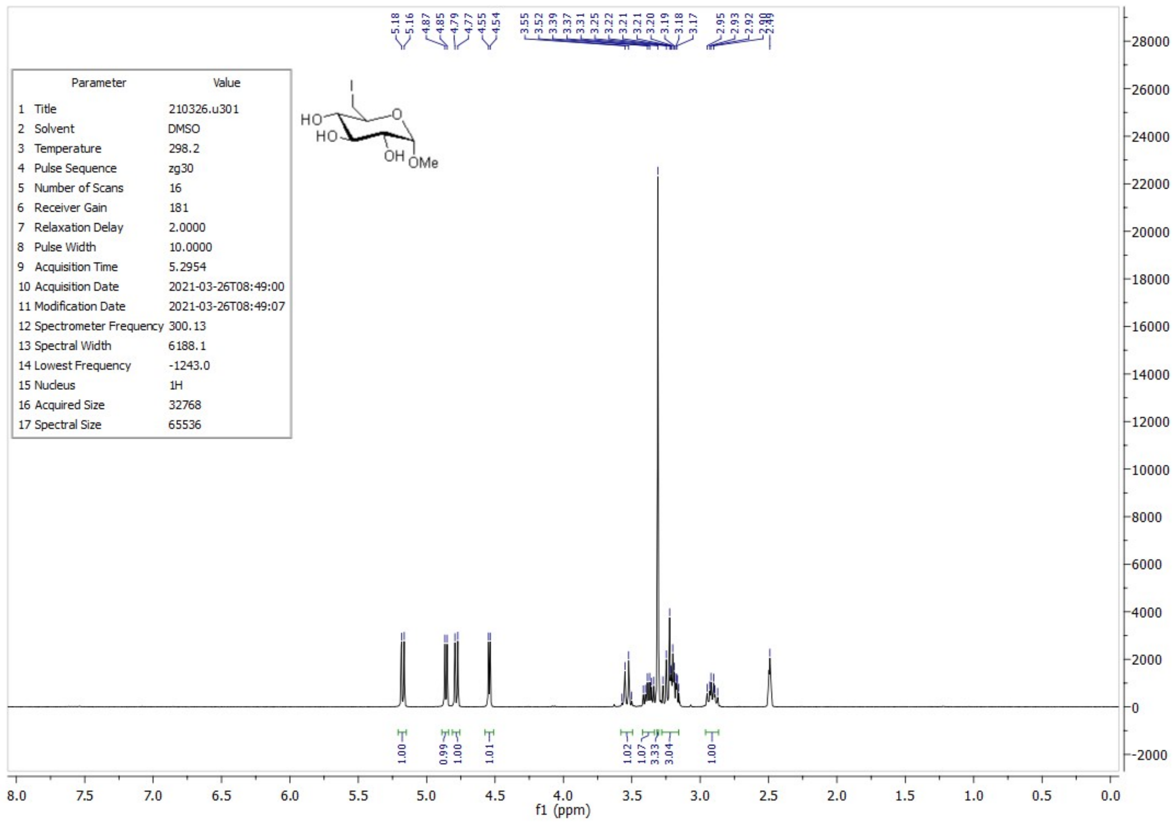

Figure S1. ^{1}H-NMR (DMSO) spectrum of compound Methyl-6-iodo-α-D-glucopyranoside **1**.

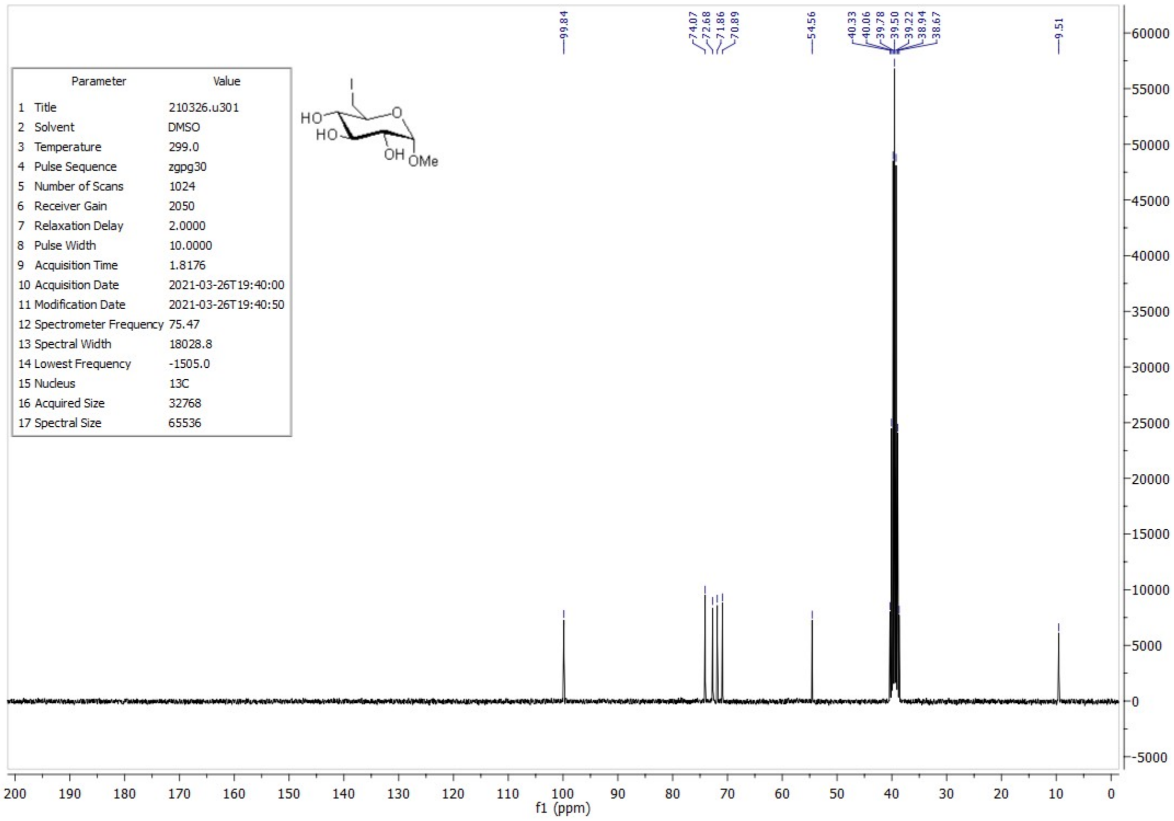

Figure S2. ^{13}C-NMR (DMSO) spectrum of compound Methyl-6-iodo-α-D-glucopyranoside **1.**

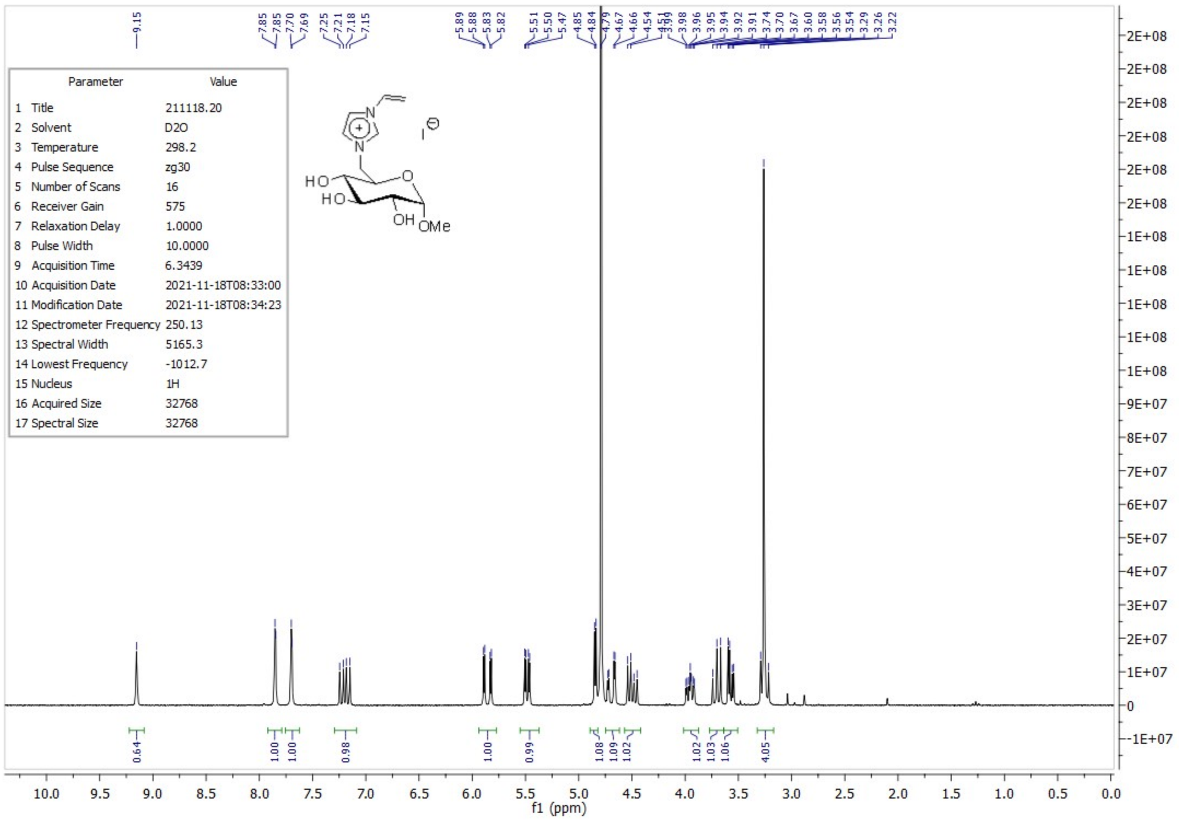

Figure S3. ^{1}H-NMR (D_2O) spectrum of compound 1-(Methyl-α-D-glucopyranosid-6-yl)-3-vinylimidazolium iodide (GVIM-I) **2.**

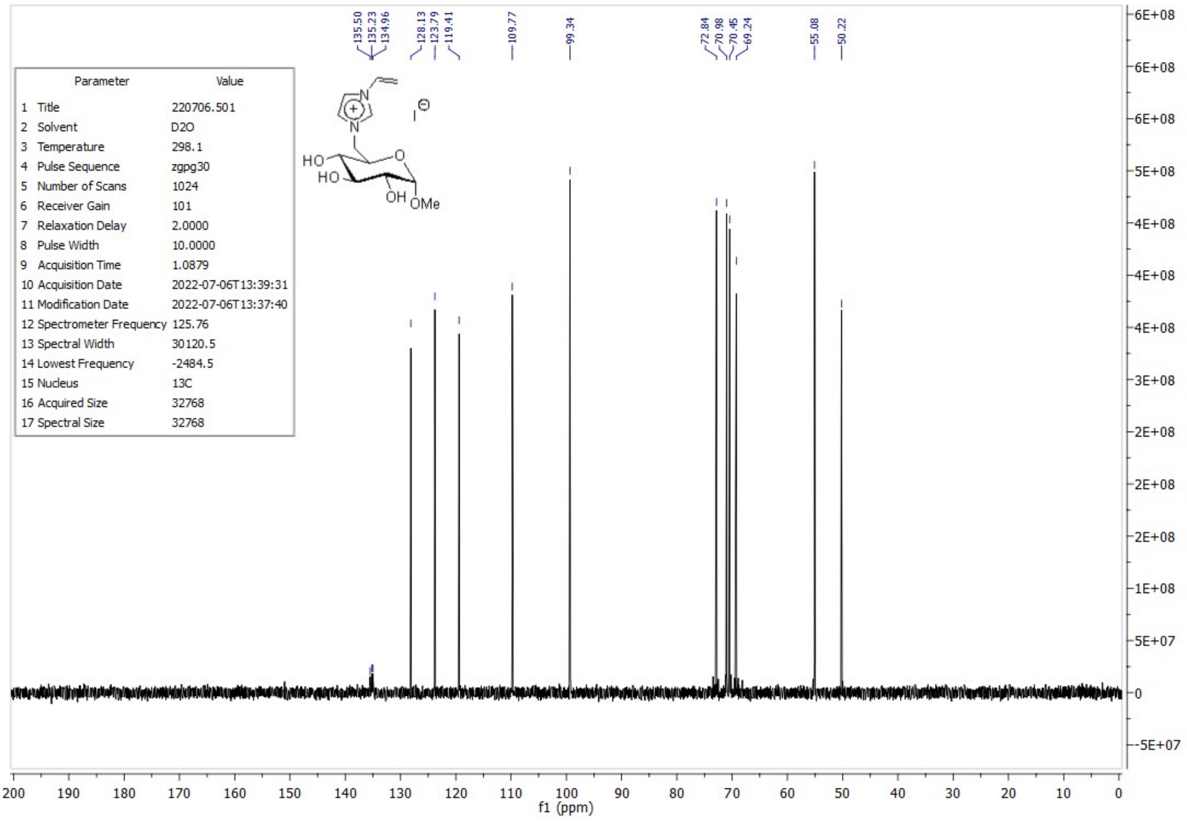

Figure S4. ^{13}C-NMR (D_2O) spectrum of compound 1-(Methyl-α-D-glucopyranosid-6-yl)-3-vinylimidazolium iodide (GVIM-I) **2**.

1.2 ATR-IR spectra

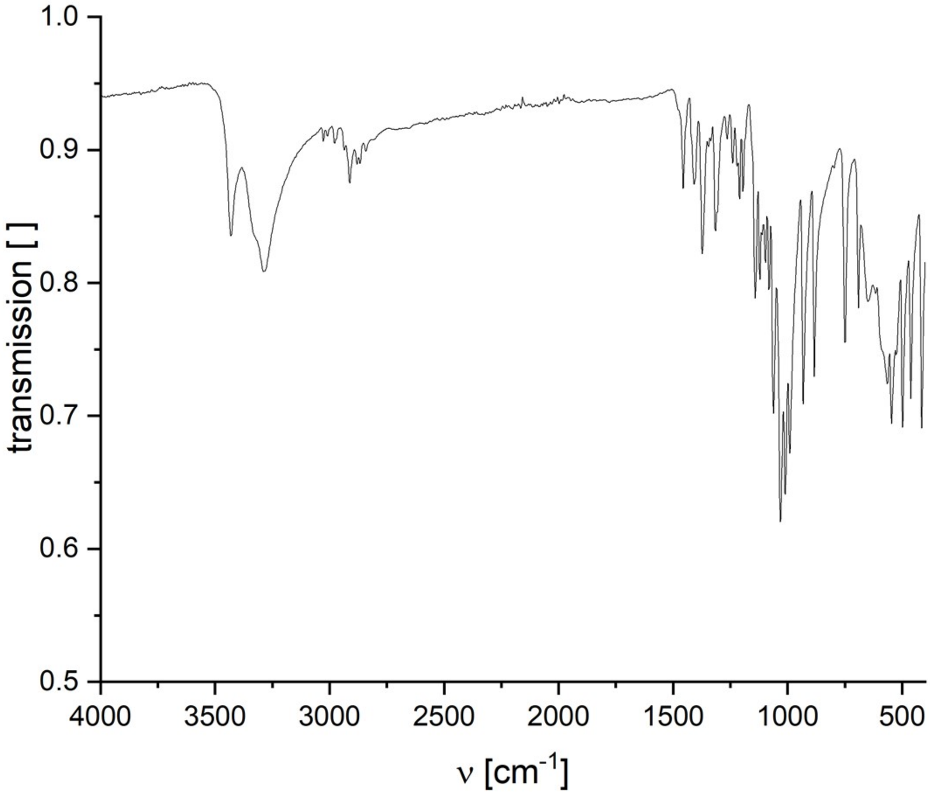

Figure S5. ATR-FTIR spectrum of compound **1**.

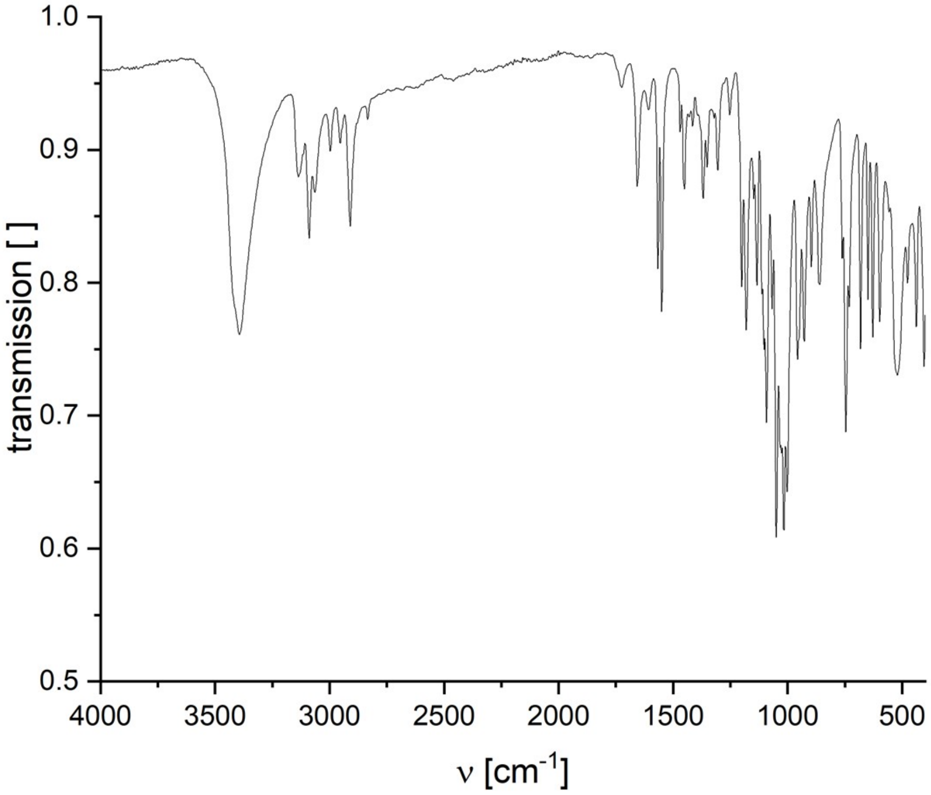

Figure S6. ATR-FTIR spectrum of compound **2**.

1.2 Weighing scales for hydrogel production

Table S1. Weighing-in scale for the production of hydrogels with LAP as initiator, according to i).

	GVIM-I		PBS	Crosslinker		LAP	
	c [mol/l]	m [mg]	V [µl]	c [mol%]	V [µl]	c [w%]	m [mg]
**EGDA**	1.25	100	200	10	3.9	3.1	9.4
**PEGDA 250**	1.25	100	200	10	5.7	2.0	6.1
**PEGDA 700**	1.25	100	200	10	15.7	0.5	1.6
**MBAA**	1.25	100	200	10	3.9	1.0	3.0
**PEGDA 575**	1.25	100	200	10	12.9	0.5	1.6

Table S2. Weighing-in scale for the production of hydrogels with APS/TEMED as initiator, according to ii).

	GVIM-I		PBS	Crosslinker		APS/TEMED		
	c [mol/l]	m [mg]	V [µl]	c [mol%]	V [µl]	c_{total} [w%]	V_{APS} [µl]	V_{TEMED} [µl]
**MBAA**	1.25	100	172.1	13.0	5.0	10.7	27.9[a]	27.9
**PEGDA 575**	1.25	100	162.5	10.4	13.4	6.8	37.5[b]	18.0

Note to APS solution: a) 400 mg in 1 ml PBS and b) 200 mg in 1 ml PBS.

2. Antimicrobial Activity – Disk Diffusion Method

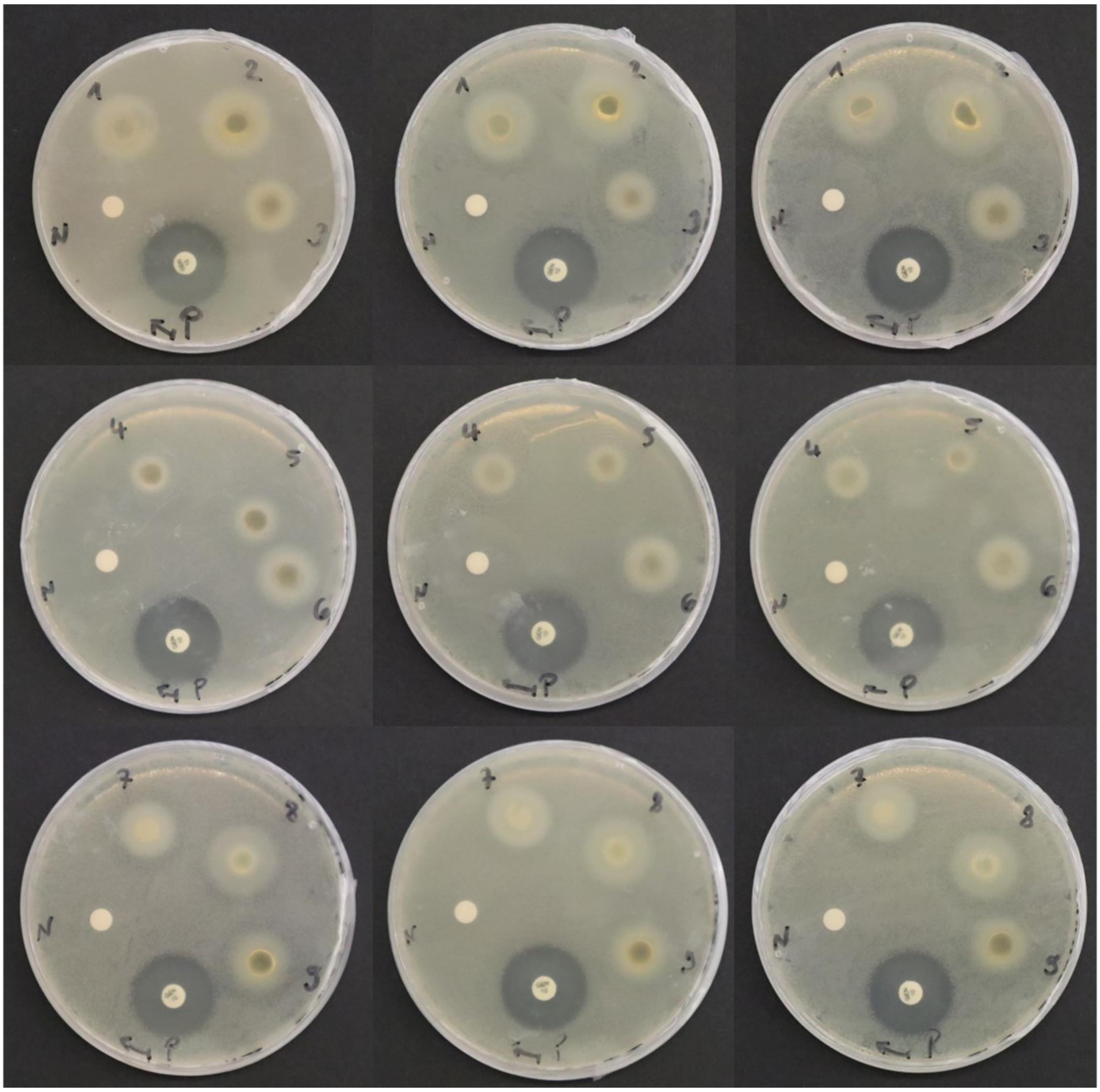

Figure S7. Overview of the disk diffusion tests against *B. subtilis* (P positive control Gentamicin; N negative control LB medium; 1 EGDA; 2 P250; 3 P575 10%; 4 P575 15%; 5 P575 20%; 6 P700; 7 MBAA LAP; 8 MBAA A/T; 9 P575 A/T).

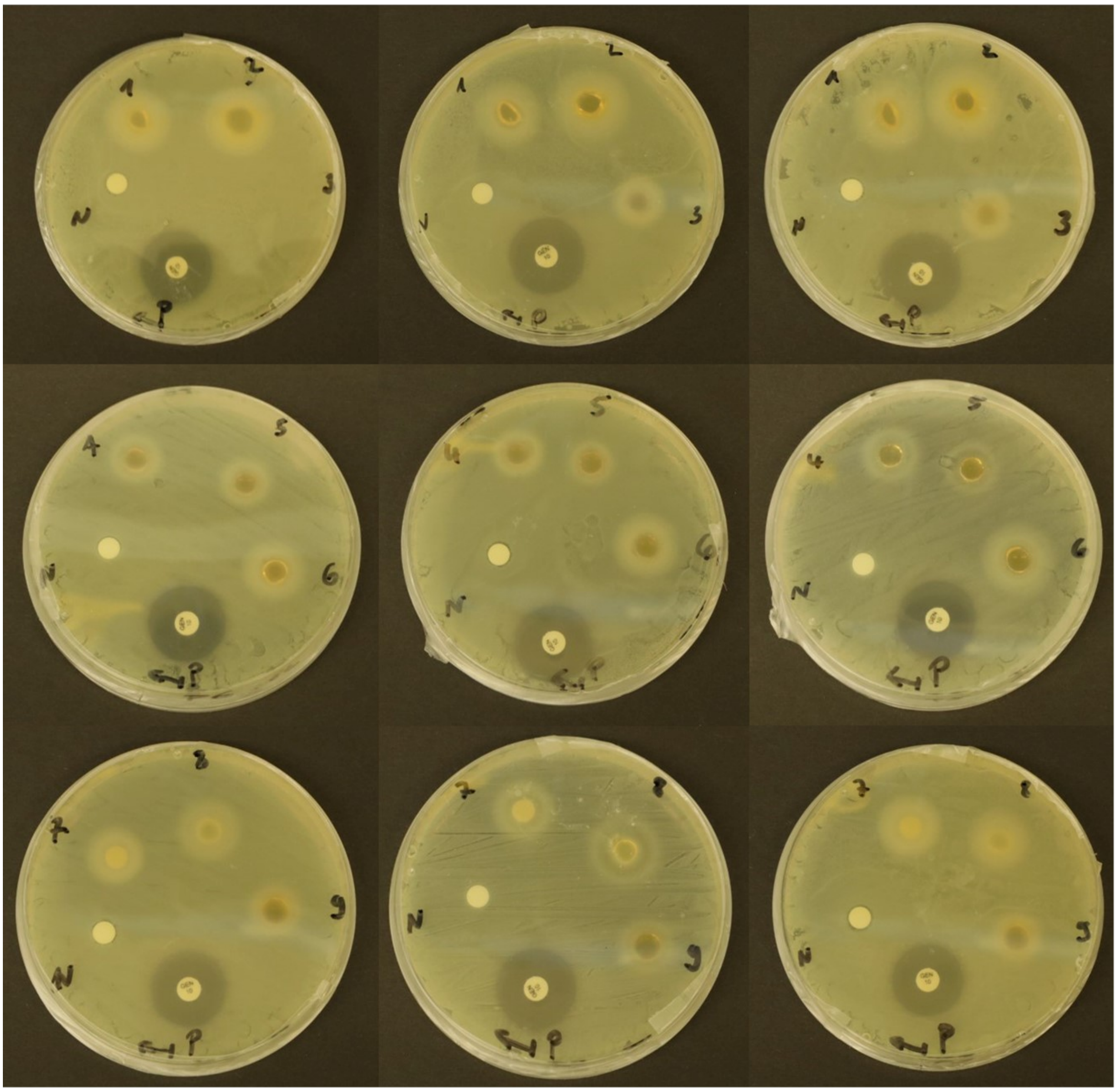

Figure S8. Overview of the disk diffusion tests against *E. coli* (P positive control Gentamicin; N negative control LB medium; 1 EGDA; 2 P250; 3 P575 10%; 4 P575 15%; 5 P575 20%; 6 P700; 7 MBAA LAP; 8 MBAA A/T; 9 P575 A/T).

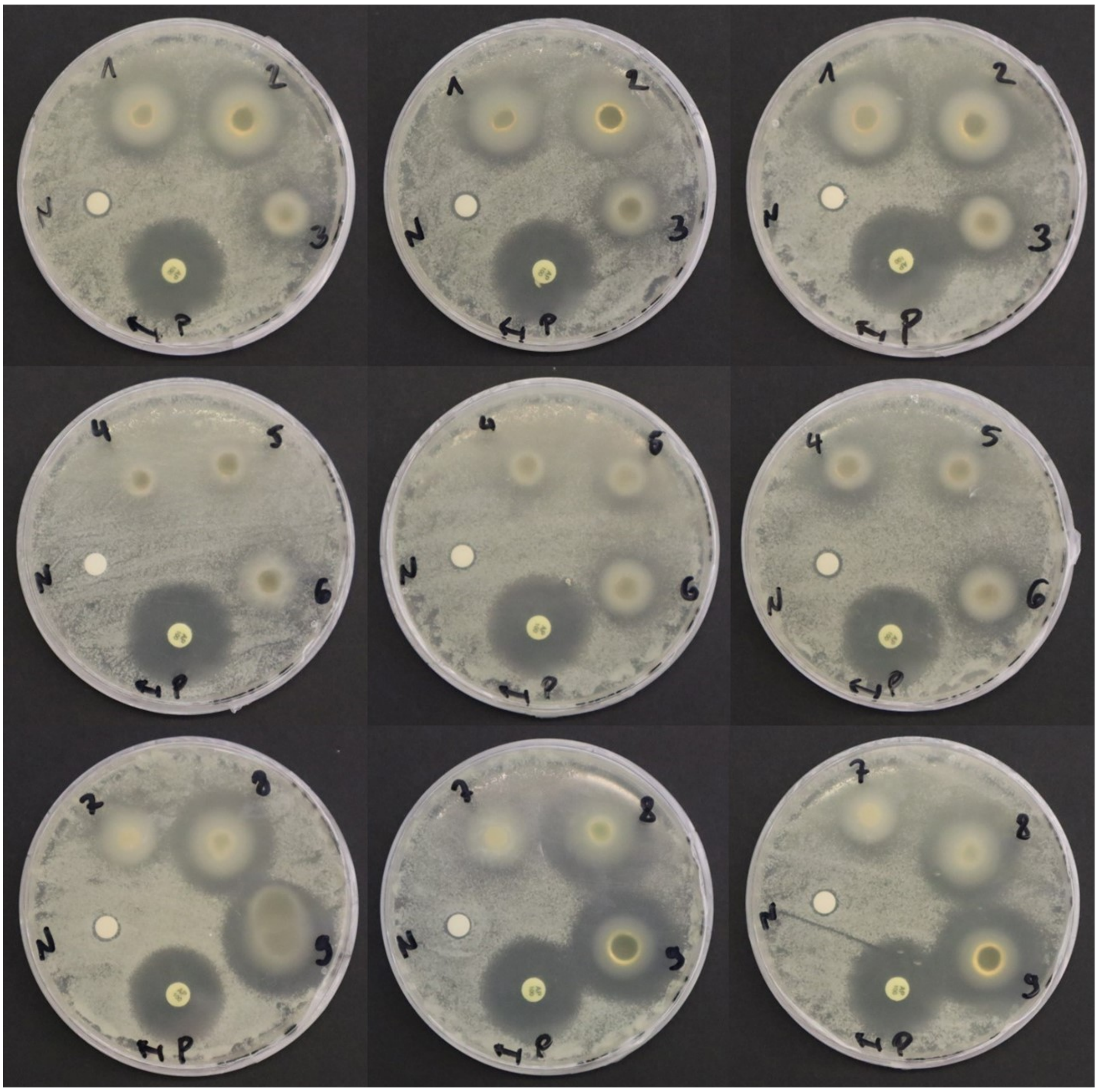

Figure S9. Overview of the disk diffusion tests against *C. aurisi* (P positive control Amphotericin B; N negative control LB medium (+ 2% glucose); 1 EGDA; 2 P250; 3 P575 10%; 4 P575 15%; 5 P575 20%; 6 P700; 7 MBAA LAP; 8 MBAA A/T; 9 P575 A/T).

3. Cell viability of L929 cells - CellTiter blue (CTB) viability assay

3.1. Eluate tests

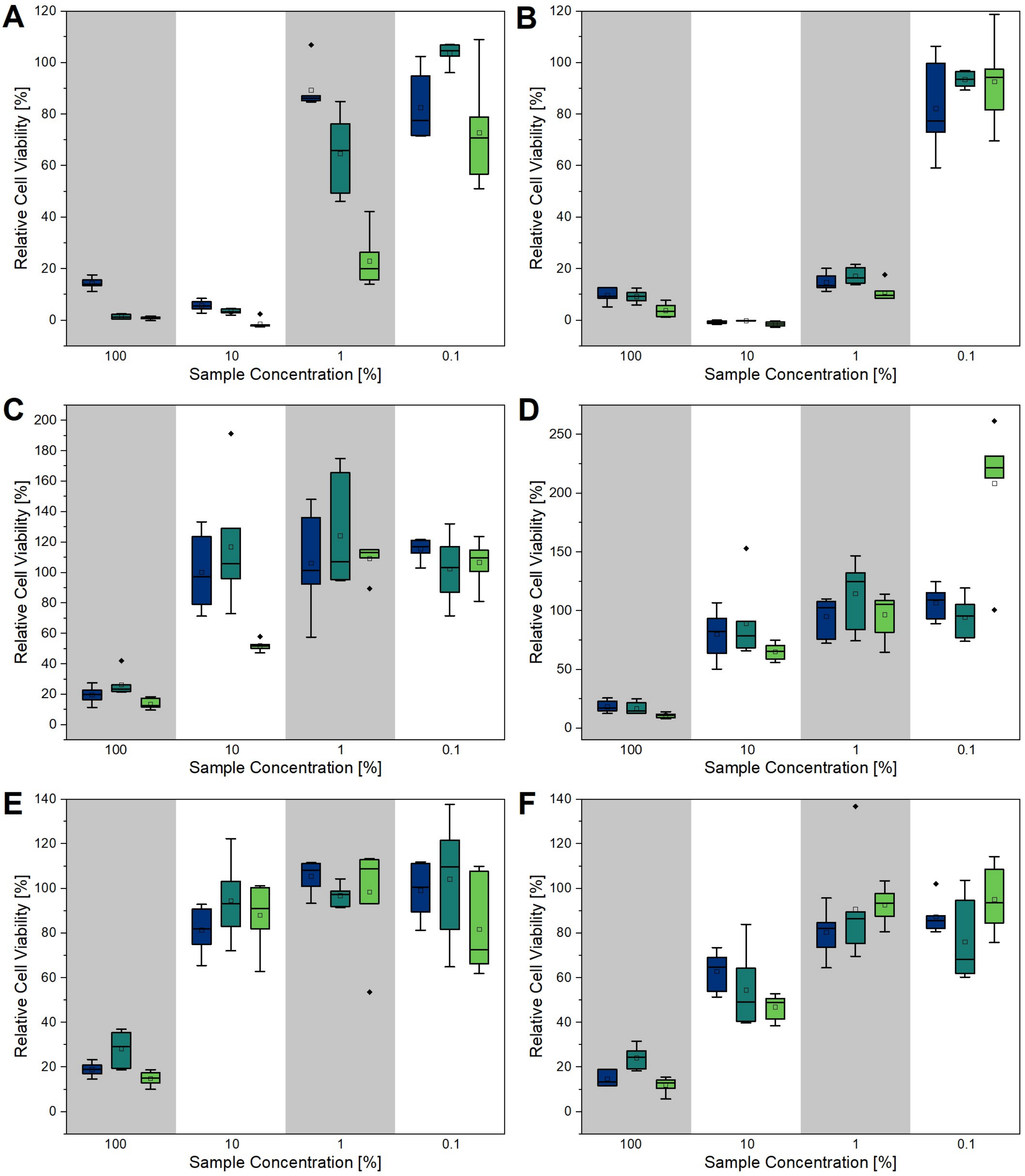

Figure S10. CTB of different hydrogel eluate samples: A EGDA; B P250; C P575 10%; D P575 15%; E P575 20%; F P700.

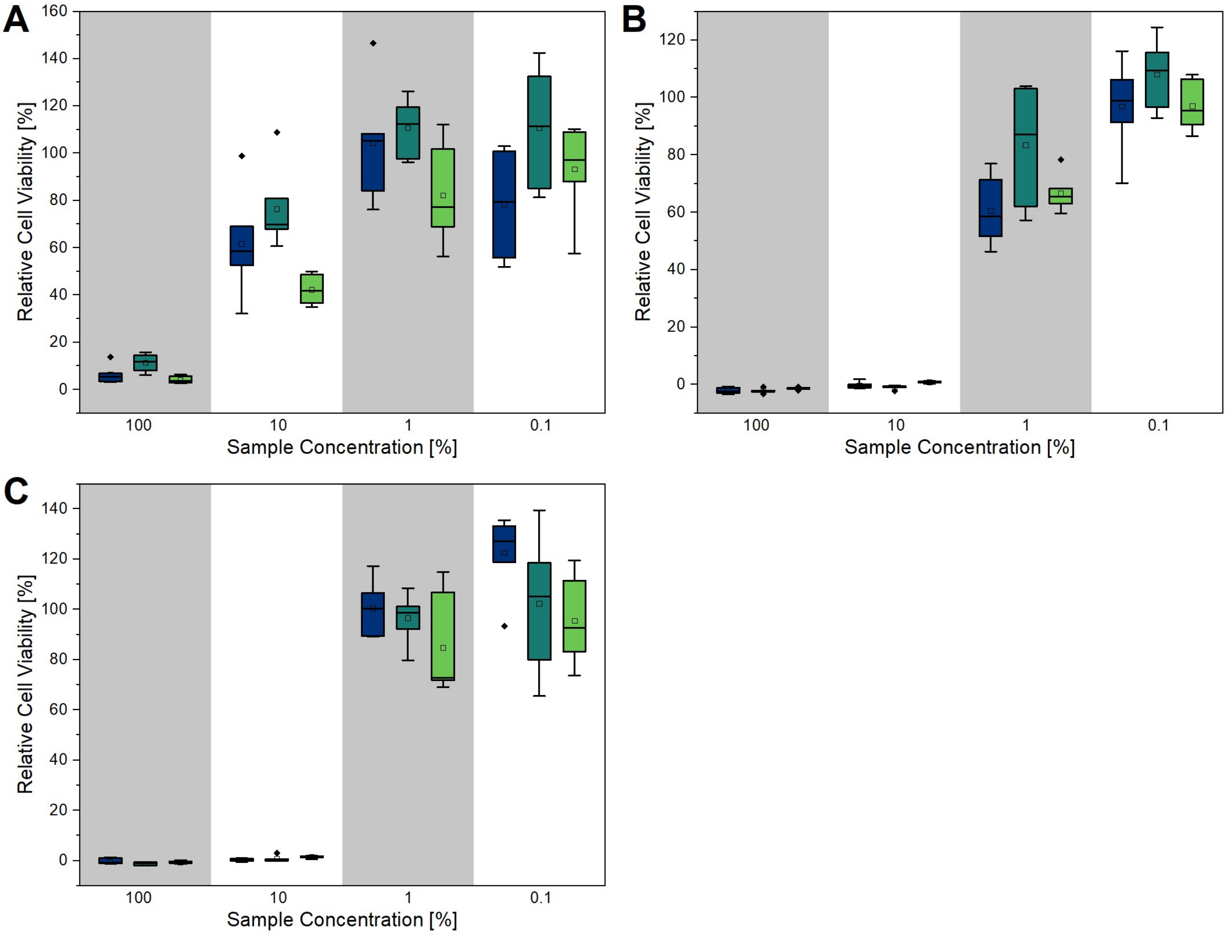

Figure S11. CTB of different hydrogel eluate samples: A MBAA; B MBAA A/T; C P575 AT.

3.2. Direct Contact tests

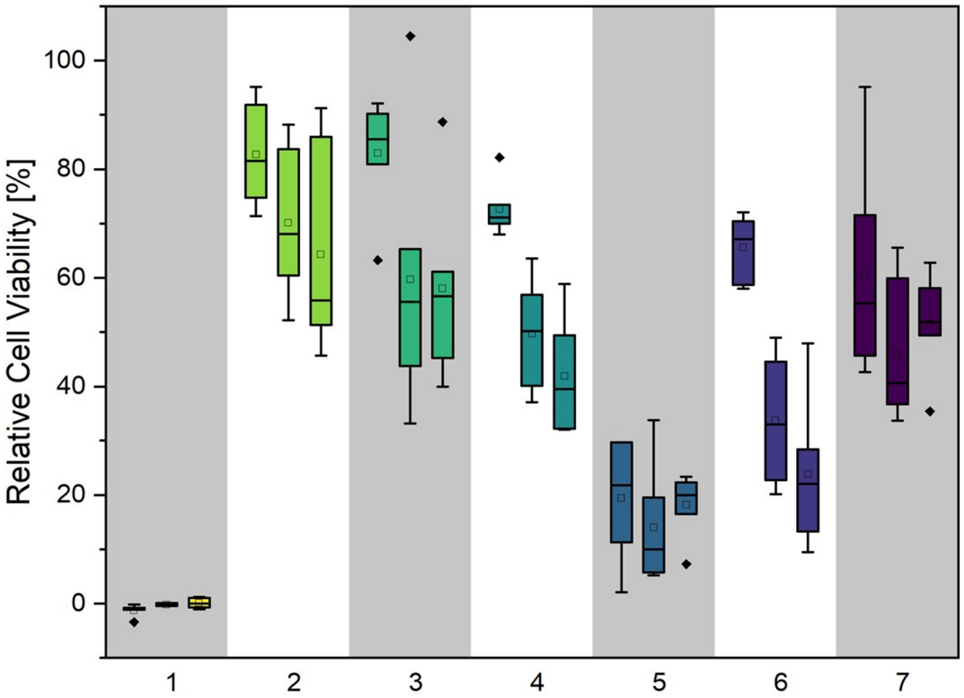

Figure S12. CTB of all 3 biological replicates of different treated hydrogels with **1** no treatment, **2** washing in DMEM 72 h, **3** washing in DMEM 3x24 h, **4** washing in EtOH (70%) 24 h and in DMEM 2x24 h, **5** UV irradiation 1 h, **6** UV irradiation 1 h and washing in DMEM 3x24 h, **7** UV irradiation 1 h, washing in EtOH (70%) and in DMEM 3x24 h.

4. Microscopic images of L929 cells

4.1. Eluate tests

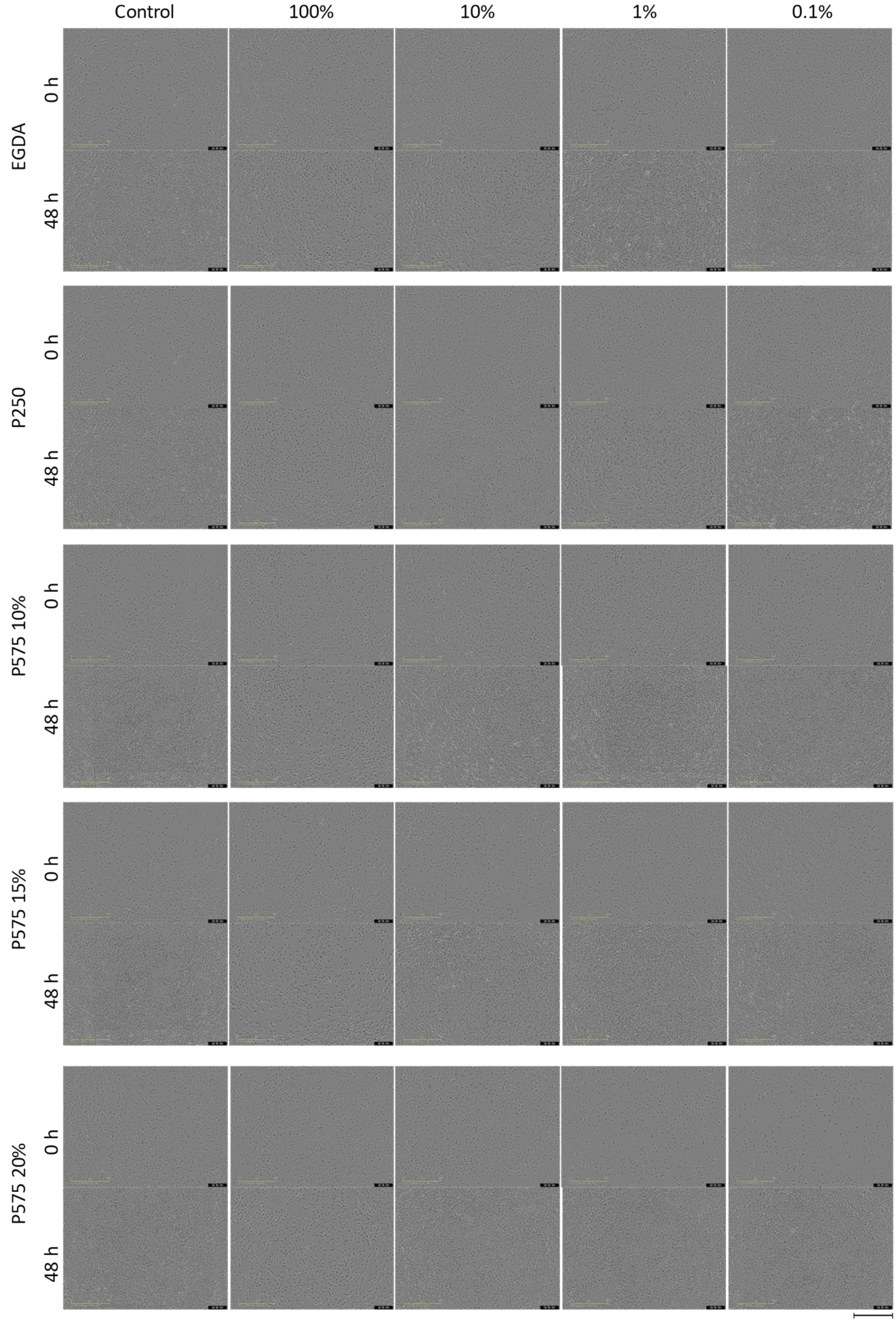

Figure S13. Microscopic brightfield images of L929 cells after 0 h and 48 h in different concentrated (100%, 10%, 1%, and 0.1%) hydrogel eluates (EGDA, PEGDA 250, PEGDA 575 10%, PEGDA 575 15%, and PEGDA 20%). Scale bar 400 µm.

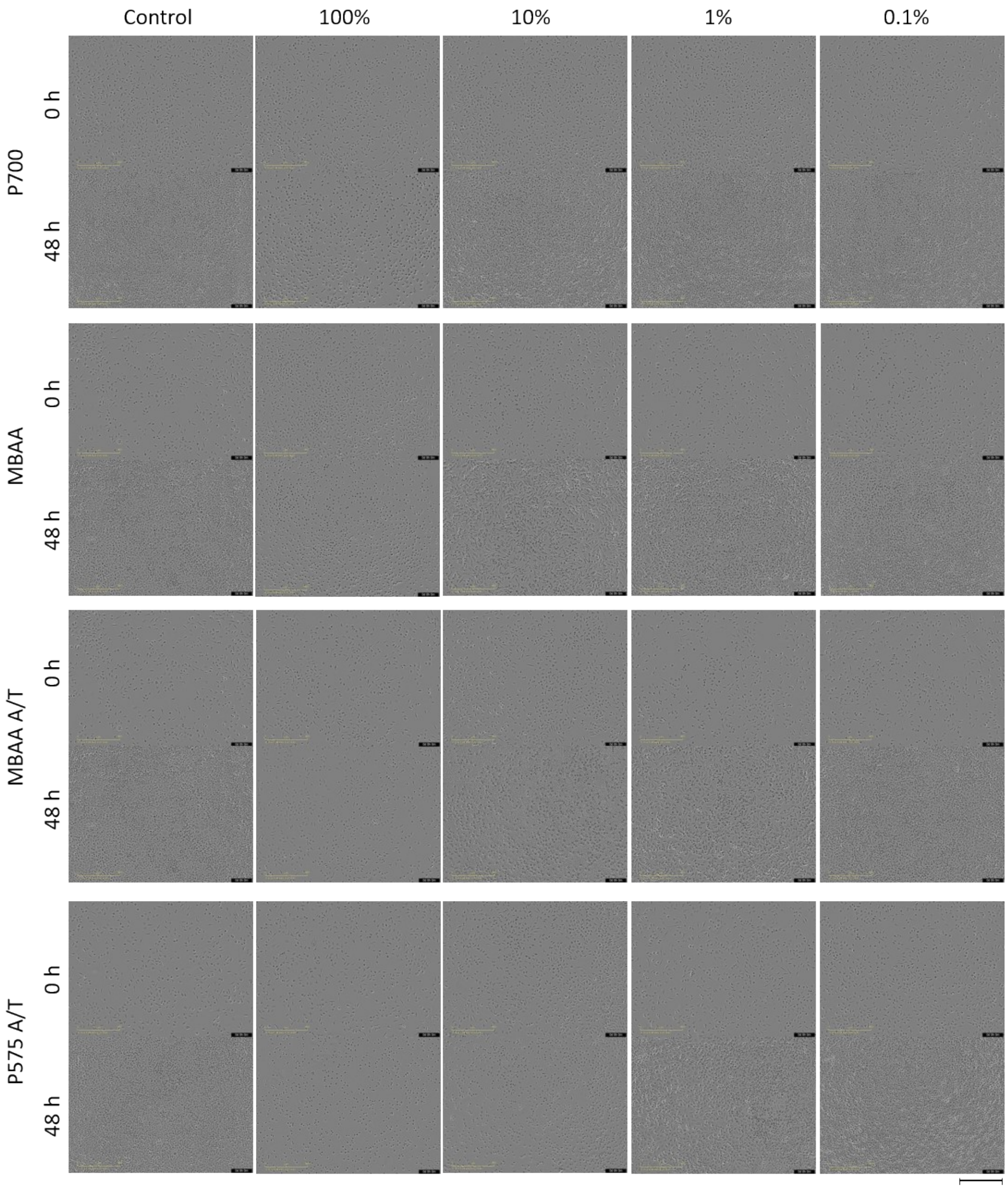

Figure S14.Microscopic brightfield images of L929 cells after 0 h and 48 h in different concentrated (100%, 10%, 1%, and 0.1%) hydrogel eluates (PEGDA 700, MBAA, MBAA A/T, and PEGDA 575 A/T). Scale bar 400 µm.

4.2. Direct contact tests

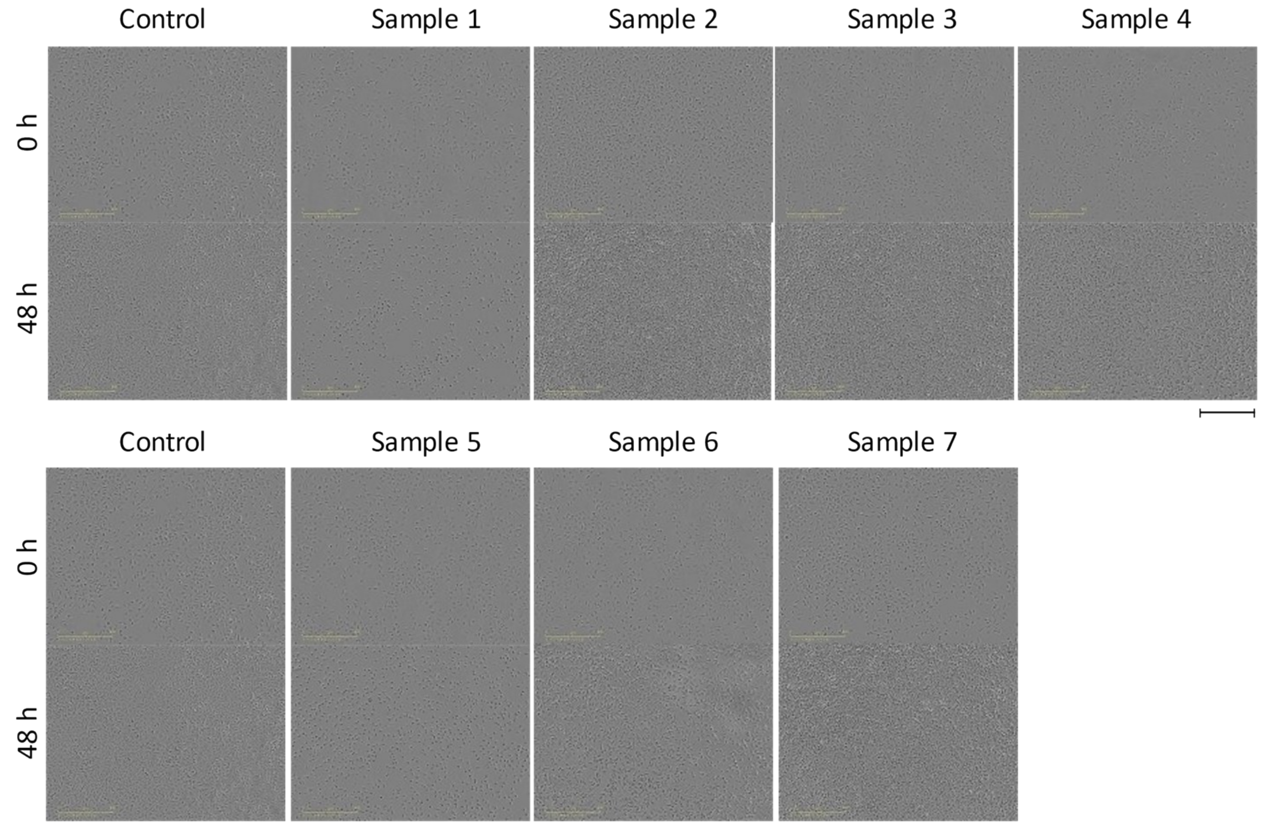

Figure S15. Microscopic brightfield images of L929 cells after 0 h and 48 h contact with different treated hydrogels with **1** no treatment, **2** washing in DMEM 72 h, **3** washing in DMEM 3x24 h, **4** washing in EtOH (70%) 24 h and in DMEM 2x24 h, **5** UV irradiation 1 h, **6** UV irradiation 1 h and washing in DMEM 3x24 h, **7** UV irradiation 1 h, washing in EtOH (70%) and in DMEM 3x24 h.

5. Cell staining (live/dead assay) - Calcein-AM/PI staining

5.1. Eluate tests

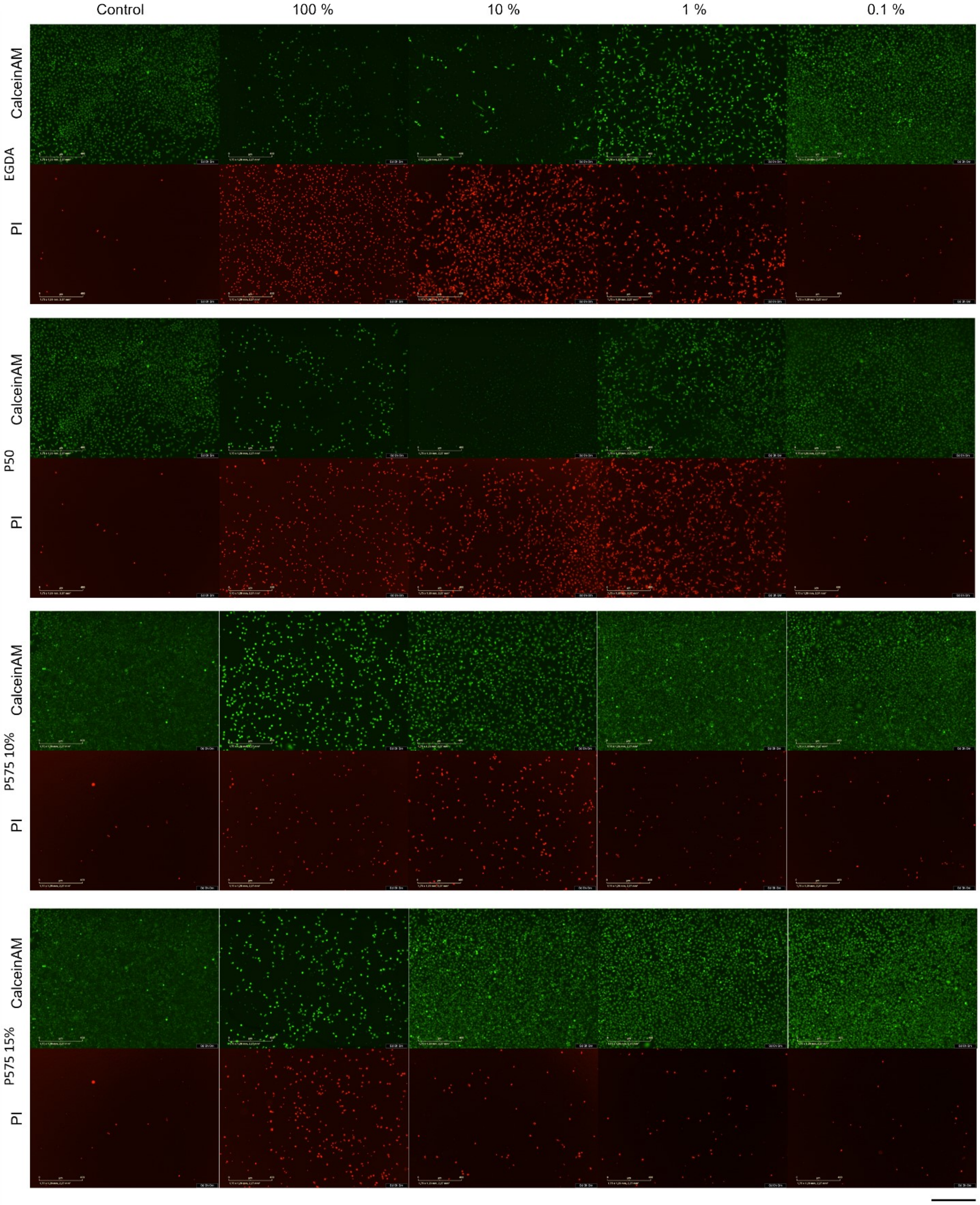

Figure S16. Calcein-AM/PI staining of L929 cell cultivated for 48 h in different concentrated (100%, 10%, 1% and 0.1%) hydrogel eluates from EGDA, PEGDA 250, PEGDA 575 10% and PEGDA 575 15% prepared with LAP (scale bar 400 µm).

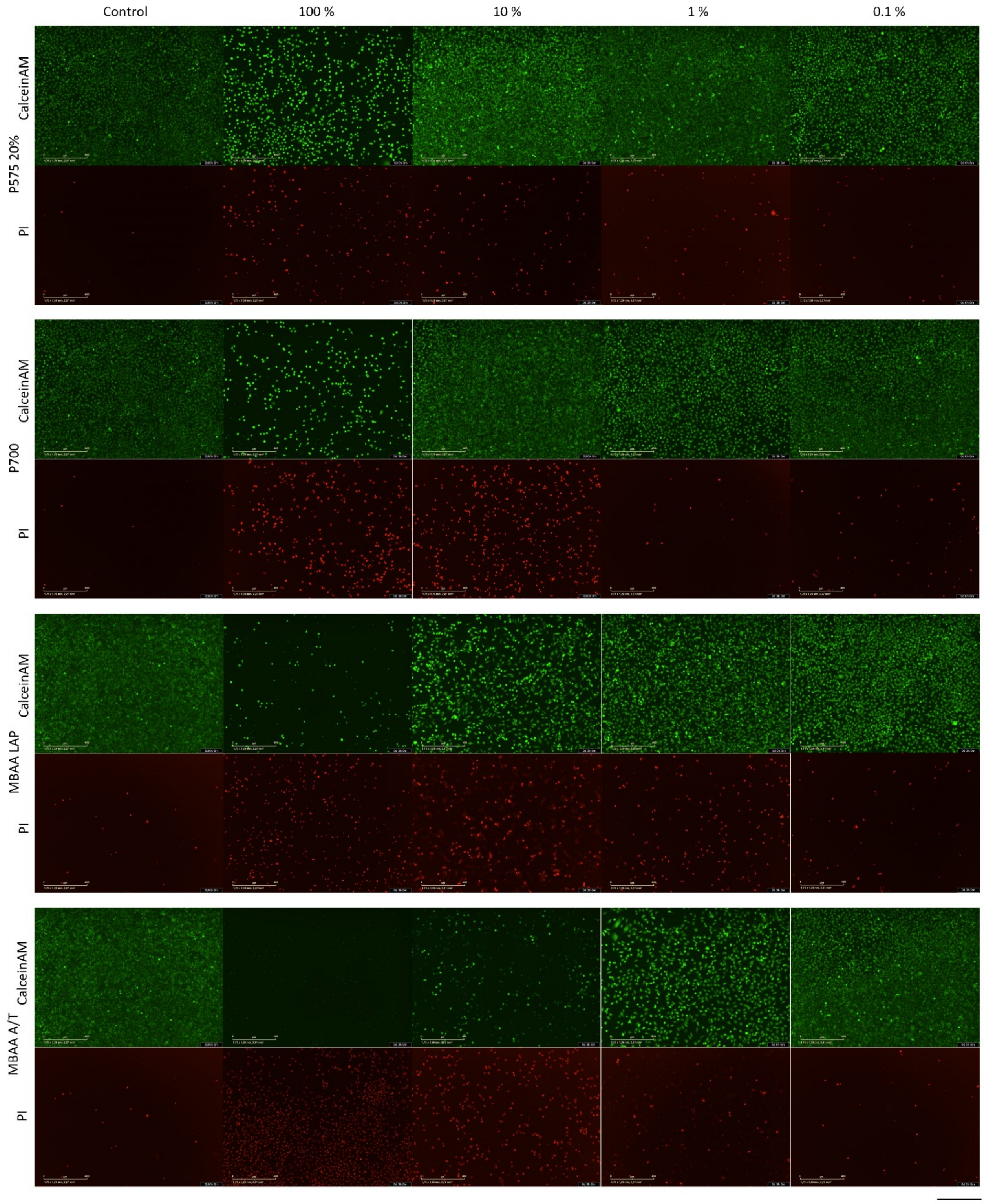

Figure S17. Calcein-AM/PI staining of L929 cell cultivated for 48 h in different concentrated (100%, 10%, 1% and 0.1%) hydrogel eluates from PEGDA 575 20% and PEGDA 700 and MBAA prepared with LAP and MBAA prepared with APT/TEMED (scale bar 400 µm).

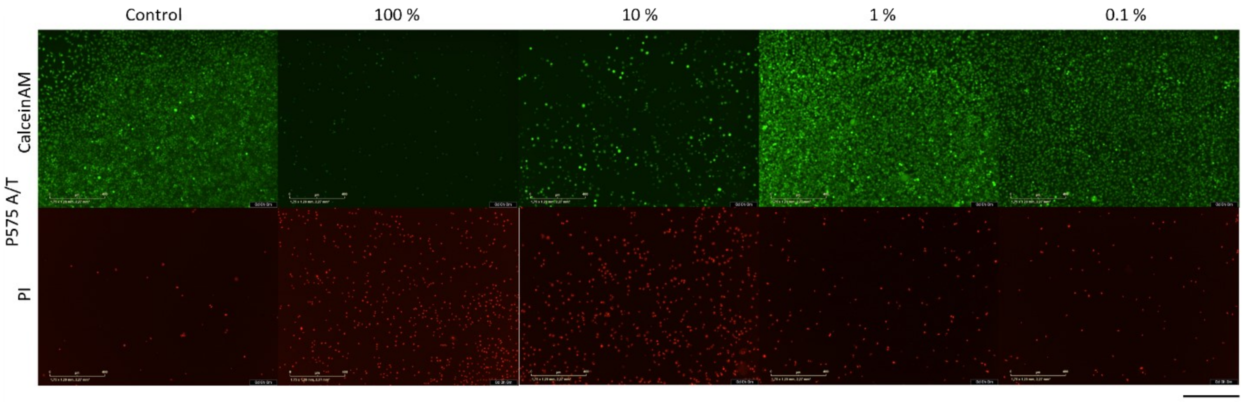

Figure S18 Calcein-AM/PI staining of L929 cell cultivated for 48 h in different concentrated (100%, 10%, 1% and 0.1%) hydrogel eluates from PEGDA 575 prepared with APT/TEMED (scale bar 400 µm).

5.2. Direct contact tests

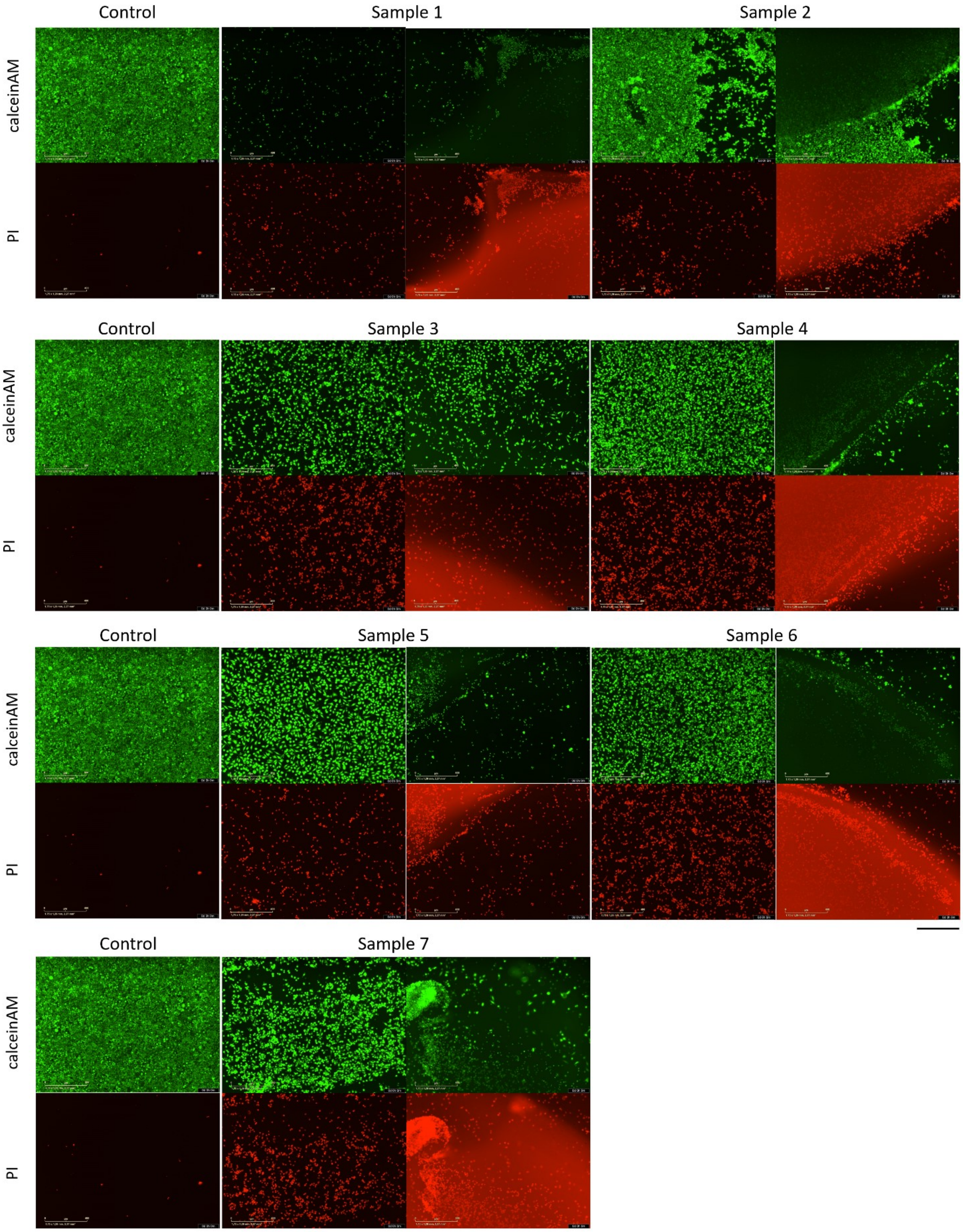

Figure S19. Calcein-AM/PI staining of L929 cell cultivated for 48 h in contact with different treated hydrogels with **1** no treatment, **2** washing in DMEM 72 h, **3** washing in DMEM 3x24 h, **4** washing in EtOH (70%) 24 h and in DMEM 2x24 h, **5** UV irradiation 1 h, **6** UV irradiation 1 h and washing in DMEM 3x24 h, **7** UV irradiation 1 h, washing in EtOH (70%) and in DMEM 3x24 h. For each sample, a picture is shown with and without hydrogel (scale bar 400 µm).

6. LC-MS

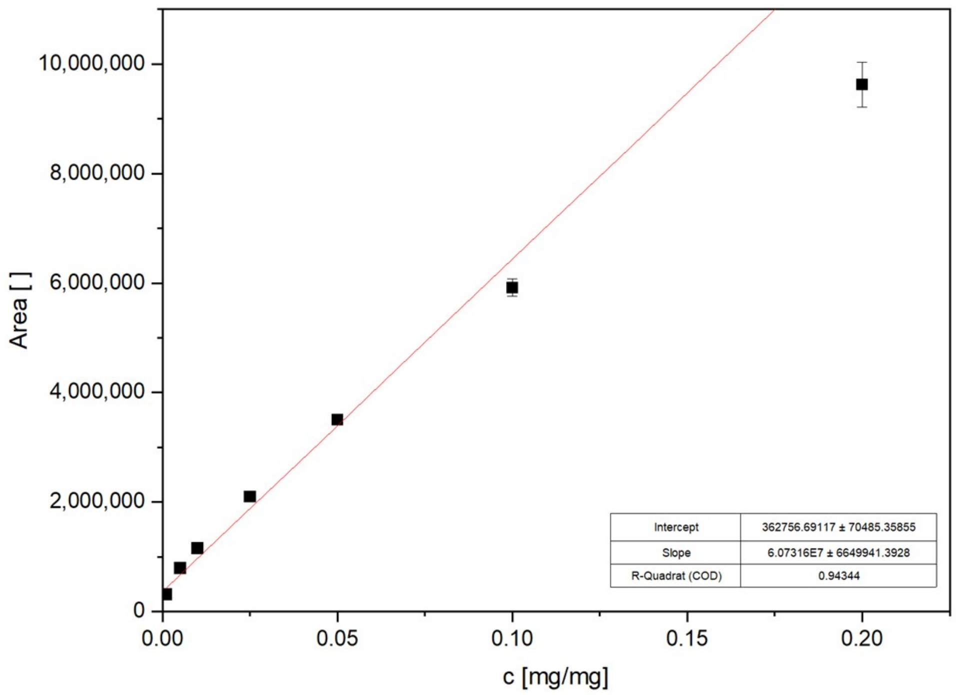

Figure S20. Calibration of GVIM-I via LC-MSMS.

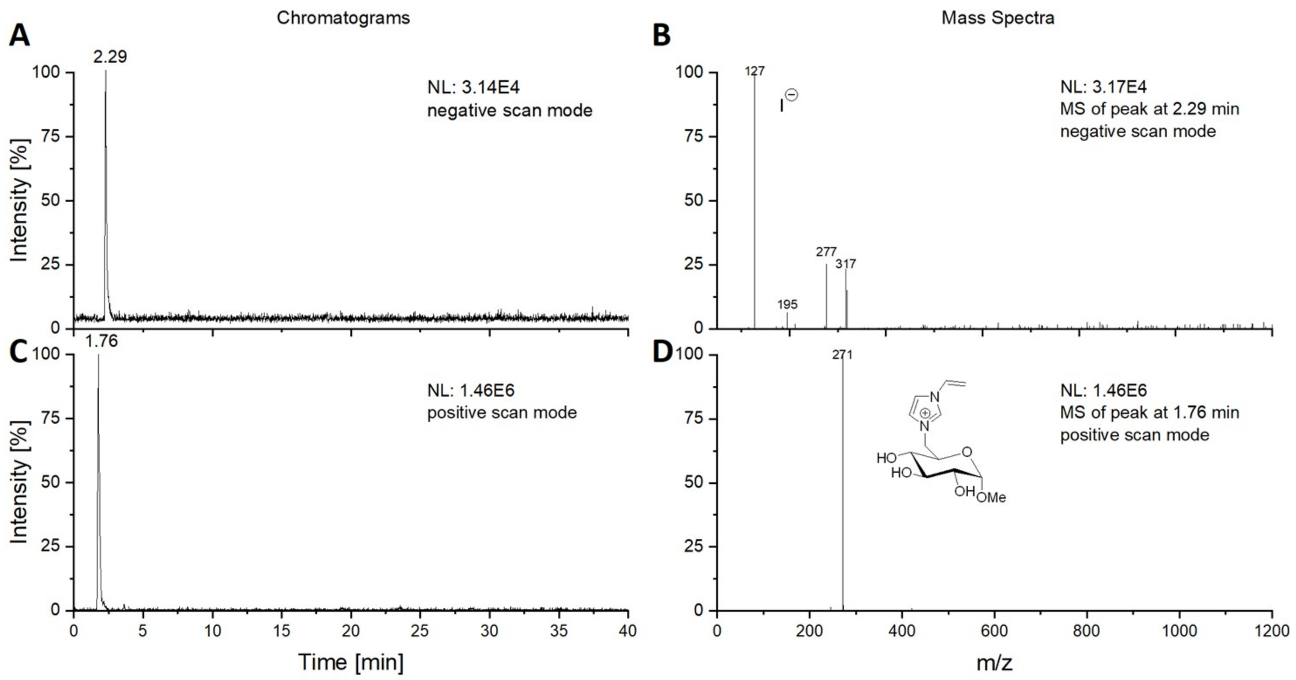

Figure S21. **A** Chromatogram of pure GVIM-I in negative scan mode, **B** Mass spectrum corresponding to A, **C** Chromatogram of pure GVIM-I in positive scan mode and **D** Mass spectrum corresponding to C.

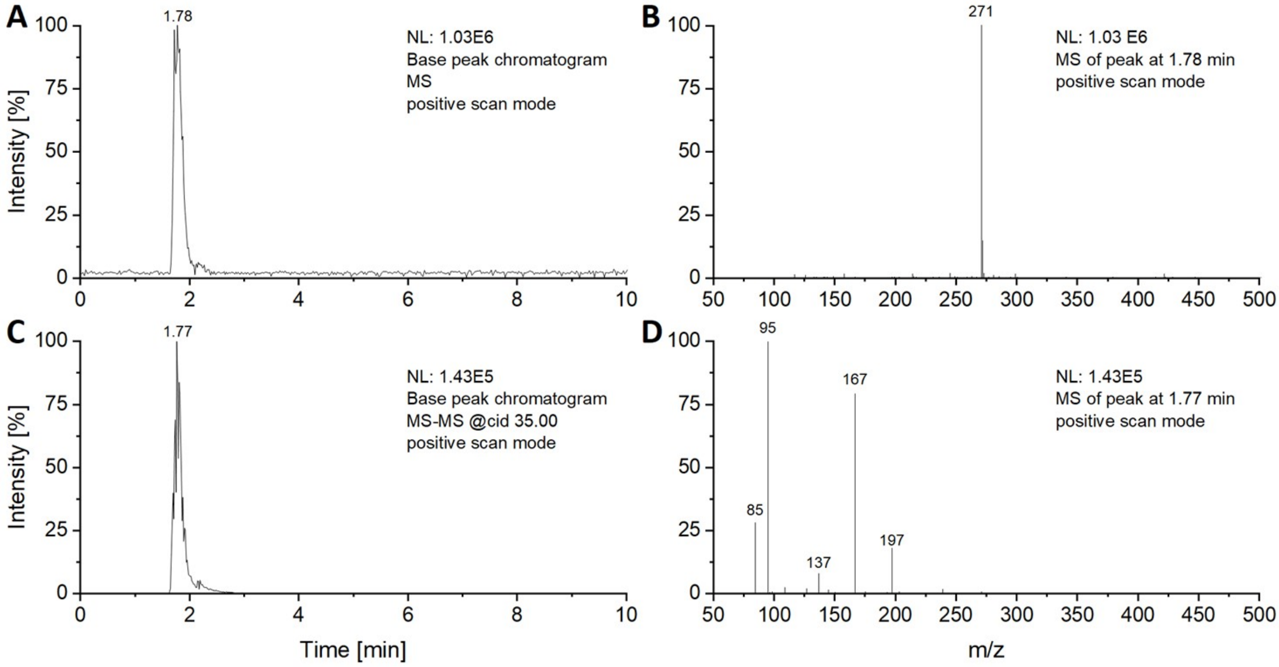

Figure S22. **A** Chromatogram of sample B1 48 h in positive scan mode (MS), **B** mass spectra (MS) of sample B1 48 h corresponding to A, **C** Chromatogram of sample B1 48 h in positive scan mode (MSMS) and **D** mass spectra (MSMS) of sample B1 48 h corresponding to C.
